# Supplementary material for: ‘Long journey to shelter’: a study protocol: a prospective longitudinal analysis of mental health and its determinants, exposure to violence and subjective experiences of the migration process among adolescent and young adult migrants in Sweden
Source: BMJ Open. 2021 Sep 30;11(9):e043822. doi: 10.1136/bmjopen-2020-043822 (PMC8487179; doi:10.1136/bmjopen-2020-043822)
Supplement: Author's manuscript [file bmjopen-2020-043822.draft_revisions.pdf]

BMJ Open

BMJ Open is committed to open peer review. As part of this commitment we make the peer review history of every article we publish publicly available.

When an article is published we post the peer reviewers' comments and the authors' responses online. We also post the versions of the paper that were used during peer review. These are the versions that the peer review comments apply to.

The versions of the paper that follow are the versions that were submitted during the peer review process. They are not the versions of record or the final published versions. They should not be cited or distributed as the published version of this manuscript.

BMJ Open is an open access journal and the full, final, typeset and author-corrected version of record of the manuscript is available on our site with no access controls, subscription charges or pay-per-view fees (http://bmjopen.bmj.com).

If you have any questions on BMJ Open's open peer review process please email info.bmjopen@bmj.com

BMJ Open

"The long journey to shelter" - study protocol: a prospective longitudinal analysis of mental health and its determinants, exposure to violence and subjective experiences of the migration process among adolescent and young adult migrants in Sweden

Journal:	*BMJ Open*
Manuscript ID	bmjopen-2020-043822
Article Type:	Protocol
Date Submitted by the Author:	15-Aug-2020
Complete List of Authors:	Mattelin, Erica; Linköping University, Barnafrid, Department of Biomedical and Clinical Sciences<br>Khanolkar, Amal R ; University College London, Institute of Child Health; University College London, GOS Institute of Child Health<br>Fröberg, Frida; Linköping University, Barnafrid, Department of Biomedical and Clinical Sciences<br>Jonsson, Linda; Ersta Sköndal Bräcke University College<br>Korhonen, Laura; Linköping University, Barnafrid, Department of Child and Adolescent Psychiatry and Department of Biomedical and Clinical Sciences
Keywords:	MENTAL HEALTH, Child & adolescent psychiatry < PSYCHIATRY, PUBLIC HEALTH

SCHOLARONE™
Manuscripts

I, the Submitting Author has the right to grant and does grant on behalf of all authors of the Work (as defined in the below author licence), an exclusive licence and/or a non-exclusive licence for contributions from authors who are: i) UK Crown employees; ii) where BMJ has agreed a CC-BY licence shall apply, and/or iii) in accordance with the terms applicable for US Federal Government officers or employees acting as part of their official duties; on a worldwide, perpetual, irrevocable, royalty-free basis to BMJ Publishing Group Ltd ("BMJ") its licensees and where the relevant Journal is co-owned by BMJ to the co-owners of the Journal, to publish the Work in this journal and any other BMJ products and to exploit all rights, as set out in our licence.

The Submitting Author accepts and understands that any supply made under these terms is made by BMJ to the Submitting Author unless you are acting as an employee on behalf of your employer or a postgraduate student of an affiliated institution which is paying any applicable article publishing charge ("APC") for Open Access articles. Where the Submitting Author wishes to make the Work available on an Open Access basis (and intends to pay the relevant APC), the terms of reuse of such Open Access shall be governed by a Creative Commons licence – details of these licences and which Creative Commons licence will apply to this Work are set out in our licence referred to above.

Other than as permitted in any relevant BMJ Author's Self Archiving Policies, I confirm this Work has not been accepted for publication elsewhere, is not being considered for publication elsewhere and does not duplicate material already published. I confirm all authors consent to publication of this Work and authorise the granting of this licence.

For peer review only

"The long journey to shelter" - study protocol: a prospective longitudinal analysis of mental health and its determinants, exposure to violence and subjective experiences of the migration process among adolescent and young adult migrants in Sweden

Authors:

Erica Mattelin[1,2,3], Amal Khanolkar[4], Frida Fröberg[1], Linda S. Jonsson[5] and Laura Korhonen[1,2,6]

Affiliations:

[1] Barnafrid, Swedish National Center on Violence Against Children, Department of Biomedical and Clinical Sciences, Linköping University, Linköping, Sweden

[2] Center for Social and Affective Neuroscience, Department of Biomedical and Clinical Sciences, Linköping University, Linköping, Sweden

[3] Save the Children Sweden, Stockholm, Sweden

[4] MRC Unit for Lifelong Health & Ageing, University College of London, London, United Kingdom

[5] Ersta Sköndal Bräcke University College, Stockholm, Sweden

[6] Department of Child and Adolescent Psychiatry and Department of Biomedical and Clinical Sciences, Linköping University, Linköping, Sweden

Corresponding author: Professor Laura Korhonen, laura.korhonen@liu.se, telephone +46-13-28 11 81.

Running title: The long journey to shelter study protocol

Keywords: Migration, adolescent, young adult, mental health, violence, resilience, experience, biomarker, longitudinal study, health inequalities, ethnicity.

For peer review only

ABSTRACT

Introduction: According to the UN Refugee Agency (UNHCR), 30 – 34 million of the 79.5 million forcibly displaced persons in the end of the year 2019 were children. Exposure to violence and mental health problems such as posttraumatic stress disorder (PTSD) are frequently reported among migrant children but there is a knowledge gap in our understanding of the complex longitudinal interplay between individual, social and societal risk- and resilience factors that impact mental health and wellbeing, quality of life, and ability to function and adapt. There is also an urgent societal need to facilitate interdisciplinary and intersectoral collaborative efforts to develop effective methods to prevent, detect and respond to the needs of the migrants. This project will study adolescent and young adult migrants in Sweden using multiple methods such as quantitative analysis of data from a prospective cohort study and qualitative analysis of data gathered from teller-focused interviews. The aim is to understand how different factors impact mental health and integration into the Swedish society. Furthermore, individual experiences related to the migration process and exposure to violence itself will be studied in detail.

Methods and analysis: Study participants will include 490 migrants aged 12-25 years recruited through social services, health care, social media and the civil society. A subset (n=160) will be interviewed again after one year. Data is collected using structured and semi-structured interviews along with saliva and hair sampling. Measures include sociodemographic data, longitudinal data on mental health and its determinants including genotypes and stress-hormone levels, access to health care and the process of migration including settlement in Sweden.

Ethics and dissemination: The Regional Ethics Board of Linköping (2018/504-32) and the National Ethics Board (2019-05473) have approved the study. Results will be made available to participants, their caregivers, professionals working with migrants, researchers and the funders.

For peer review only

ARTICLE SUMMARY

Strengths and limitations of this study

- This is the first prospective longitudinal study in Sweden on newly arrived refugees and asylum seekers and aims to investigate exposure to violence and mental and physical health using face-to-face interviews.
- Using a multimethod approach including analysis of genotypes and biomarkers, the study will provide a comprehensive set of data that allows a nuanced analysis of within immigrant population as well as context- and time-dependent factors that explain the different outcomes.
- Results can potentially improve care of migrants and aid in developing current intersectoral procedures including public health policies.
- Overrepresentation of study participants from particular countries could lead to selection bias.
- Loss to follow-up could impact the longitudinal analysis.

For peer review only

INTRODUCTION

Today millions of children are fleeing their countries, either voluntarily or being forced to. The group of migrant children is heterogeneous and includes asylum-seekers and refugees fleeing with their families as well as unaccompanied minors.[1] Most refugees/asylum seekers are forced to flee their homes due to acute threats such as conflicts, famine and instability but the migration problem also reflects massive socioeconomic inequalities that exist between different countries and regions.[2 3] Faced with this reality, migration is considered to be one of the greatest complex and current challenges that cannot be tackled quickly by simple measures.[4]

In 2019, of the 79.5 million people forced to flee their homes, 40% were minors.[2] During the peak immigration year of 2015, 70,384 children applied for asylum in Sweden; about half of them were unaccompanied.[5] Thereafter, the number of migrant children in Sweden has gradually decreased reflecting the European Union policy to limit migration.[5 6]

The definition of international migration encompasses the process of leaving the home country and settlement and integration into the society of destination. In practice, the process is most often intervened with complicated, stressful and potentially hazardous transient phases such as travelling in the hand of illegal smugglers, staying in multinational refugee camps or asylum accommodations and multiple replacements between different countries, accommodations and administrative systems with subsequent interruptions in established human relationships. Thus, migration is associated with several considerable risks such as being targeted by illegal smugglers and other types of crimes,[7] exposure to potentially traumatizing events as well as mental and physical health problems[8-11] including multimorbidity[12] and premature death.[13]

The arrival of migrants may be challenging for the host country. Barajas-Gonzalez et al [14] showed that immigration enforcement in the US leads to severe effects on children´s mental health, such as children living with a constant fear of their parents being taken into custody; leading the authors to suggest that the immigration enforcement may become a form of psychological violence. But in general, it is largely unexplored what damage is caused to children by slow and insecure bureaucracy.

Current state-of-the-art has shown that migrant children have experienced potentially traumatizing events more often than non-migrant children in high-income countries. Studies

also report a higher prevalence of mental health problems, such as PTSD, anxiety, and depression, among migrants compared to non-migrant children.[8 15 16] However, the link between potentially traumatic events and mental health among migrant children has not been fully explained. This is largely because most studies till date have been cross-sectional in design, with small sample sizes and have focused on children that have already been in the host country for a considerable time.

Research literature has largely focused on risk factors and mental illness.[17] Less attention has been paid on individual and social resilience factors and their interplay with risk factors which is important for, among other things, a favorable adaptation to a new environment. Resilience entails the achievement of desirable social and emotional adaptation, although the individual was exposed to great risk.[18] Knowing that exposure to repeated violence is not only a direct danger to a child but also may have long-term consequences for the mental and physical health[19 20], efforts should be taken to strengthen the child's and family's resilience.

Objectives

The overall research aim is to study and understand how different factors impact health, wellbeing and integration of migrants in Sweden. We hypothesize that the refugee and asylum-seeking group of children is heterogeneous including well-adjusted individuals with good health and those with severe problems, multimorbidity and functional impairments. This study aims:

- To identify individual-related factors that impact health and wellbeing. Includes studies on traumatization, mental health problems, resilience as well as genotypes, stress hormones and other biomarkers from collected biomaterial.
- To study which social factors that impact health, wellbeing and integration. Includes analysis of network and support.
- To describe children´s experiences related to the entire migration process.

METHODS AND ANALYSIS

Design and participants

This study includes prospective longitudinal and cross-sectional designs. The term ’migrant’ *(“på flykt” in Swedish)* refers in this study to asylum seekers, refugees, family migrants, quota refugees and undocumented migrants. The population recruited to the study is migrant adolescents and young adults residing in Sweden during 2018-2021. As this is a

heterogenous group, 4 different study groups will be included involving participants recruited from the community as well as from clinical settings, see Figure 1.

1 *Adolescents (quantitative study):* 80 unaccompanied and 80 accompanied adolescent migrants that will be recruited in 2018 - 2021. Inclusion criteria: migrant, 12 – 18 years of age, and should not have applied for asylum in Sweden more than 6 months before the study interview.

2 *Adolescents (qualitative interviews)*: 30 adolescents for whom the inclusion criteria are 15-18 years of age, migrant, applied for asylum in Sweden at a maximum of 18 months before the interview date.

3 *Young adult migrants and Swedish born peers (quantitative study)*: This includes 100 young adults who migrated to Sweden after 2015. Inclusion criteria: migrants between the ages of 18 – 25 years living in Sweden. A comparison group consists of a sample of 100 young adults born in Sweden.

4 *Clinical sample (quantitative study)*: This will include 100 adolescent and young adults to be recruited from a trauma unit at a Refugee Medical Center in Norrköping, Sweden. Inclusion criteria: 15 – 25 years of age, migrant and living in Sweden.

Sample size

The aim is to collect data on 490 children and young adults. This sample size allows proposed analyses of primary outcomes with statistical strength of 0.80 and α <.05 and is based on previous studies with similar methodology [21 22 23] which included 95 - 138 adolescent migrants, with 47 - 77 adolescent migrants at follow-up assessments. We aim to have a larger sample to be able to do more detailed analyses.

Recruitment

Participants are recruited across Sweden since refugees/asylum-seekers are given a random municipality placement while waiting for a decision on their application for asylum. Recruitment is done via health care, social services, social media and the civil society such as non-governmental organizations working with the migrants. The clinical sample is recruited from the Refugee Medical Centre/Region Östergötland and the community comparison group mainly through high schools.

For peer review only

Recruitment of study participants is mainly via people working directly with refugee and asylum seekers who are given leaflets describing the study. Those who agree to participate will be subsequently contacted by the researchers to first obtain informed consent as well as to schedule an interview. All information is given in oral and written form in the participant`s native language.

Each interview is scheduled to last for two hours and a certified translator is used if needed. Interviews are held at a location chosen by the interviewee and/or the family.

Interviews

Semi-structured, translator assisted interviews are conducted to collect socio-demographic data on country of birth and self-identified ethnicity, family situation and schooling (see Table 1). Symptoms of mental disorders are assessed using the diagnostic interview MINI-KID/MINI.[24] Presence of PTSD is evaluated using the Child and Adolescent Trauma Screen [25] for those under 18 and PCL-5 for those over 18.[26] The Juvenile Victimization Questionnaire (JVQ) is used to measure the number of potentially traumatizing events.[27] The questionnaire has been adapted to this study to specifically assess the timing by including the question on whether potentially traumatizing events happened before, during or after the flight and additional questions on issues related to natural disasters, accidents, medical procedures, death of close relatives, poverty, separation from their parents, being captured or imprisoned and human trafficking. Resilience is evaluated using the Adolescent Resilience Questionnaire[28] including five different domains: self, family, peers, school and society. Participant will do a self-rating on physical health and well-being using the WHO-5 wellbeing index.[29] The participants' functional capacity is mapped with the GAF and WSAS.[30 31] The interviews are verified by an experienced clinical psychologist to ensure good quality. Also, a random selection of 10% of the interviews are quality assessed independently by two members in the research group.

The qualitative interviews are conducted using the "Teller-focused interview" method which is particularly suitable for studies on sensitive topics and ongoing processes.[32] The interviews are semi-structured and include few overarching questions that covers areas such as the adolescents' experiences in the home country (e.g. school, friends, hobbies, wellbeing), issues related to the entire migration process (e.g. reasons to flee, memories from the journey,

exposure to violence if any), life during the first 18 months in Sweden and their future aspirations for the 10 years ahead. Total time per assessment is approximately 60 minutes.

Biological material

A 3cm long hair sample is collected and stored at room temperature until analysis. Saliva samples are collected using Salivette Cortisol (Sarstedt AG & Co. KG, Nurnbrecht, Germany) and stored at -80° until analysis.

Data-analysis plan

Figure 2 provides an overview of the main exposures, outcomes, confounders and analysis that will be conducted. Descriptive analysis of collected data will include presenting continuous variables as mean values with standard deviations and categorical variables as proportions for the entire study sample but also by important covariates like sex, unaccompanied/accompanied and country of origin. Associations of interest between various variables will be analyzed using univariate linear regression or chi-square tests for differences of proportions for continuous and categorical variables, respectively.

A series of regression models will be performed to study associations between exposures (e.g. exposure to violence, asylum status) and outcomes of interest (e.g. mental health, functional ability, comorbidity (for example co-occurrence of mental and physical health conditions) using multivariate linear and logistic regression for continuous and categorical outcomes respectively. Each set of regression models will be adjusted for potential confounders such as sex, family socioeconomic position, age and country of origin.

An analysis of individual, environmental and social factors that explain better adaptation, resilience, and level of function will be conducted. First, a descriptive analysis of the sociodemographic background, exposure to violence, the prevalence of mental and physical health problems, wellbeing and resilience factors, in the adolescent cohort and young adult sample will be performed. Second, the prevalence of violence, and mental health problems, and the association between violence and mental health problems in the different phases of the migration process in both groups will be estimated. Besides, the potential moderating effect of resilience factors on the association between exposure to violence and mental health problems in the adolescent cohort will be analyzed. Third, because it is assumed that the migration and resilience processes, and the development of mental health problems, are complex and

For peer review only

potentially non-linear, multidirectional and multilevel, we will also conduct a multilevel analysis of longitudinal data.

Qualitative interviews will be analyzed according to the principles for thematic analysis.[33] Genotypes and biomarkers such as stress hormone levels and immunoprofiles will be analyzed using standard methods.

Gender aspects

We know, based on previous research that the associations between certain risk factors, such as traumatic events, and mental health differs between the sexes.[34] Such differences are often attributed to genetic or biological factors, and sociological processes, such as conforming to gender normative behavior or how girls and boys are educated differently, for example. When it comes to the experiences and health of migrant children, less effort has been made to understand the importance of gender. In our study, we will initially stratify analyses by sex and potential sex differences will then be interpreted with a gender perspective; with the intention of understanding and explaining the importance of gender in the link between exposure and outcome. We will also take into account that our respondents may choose to identify themselves by ethnicity, sexuality, family socioeconomic background.

Methodological issues

The longitudinal study design will allow analysis of changes over time in the same study participant, providing stronger evidence for causality than could be obtained from a cross-sectional design. Combined interview and biological data will provide a versatile set of information that can be used to address different kinds of research questions. Use of standardized structured and semi-structured interviews and validated instruments will also allow comparisons to other studies.

The primary limitation of this study may arise if migrants from a few countries with a high certainty of getting asylum such as Syria will be overrepresented in the study. Furthermore, we could also have the risk of concerned parents or social workers not letting all the adolescents participate because of fear of causing distress. This may exclude certain groups such as those with existing mental health problems and/or exposed to violence.

For peer review only - http://bmjopen.bmj.com/site/about/guidelines.xhtml

Yet another potential limitation is the loss to follow-up because migrants might leave the country. Efforts to minimize loss to follow-up will include respecting the time commitment of patients, formal consented tracking procedures of patients enrolled, multiple contacts for arranging follow-up and flexible hours and places for interviews.

Data management, storage and security

Throughout the course of this project, research data is collected and processed according to FAIR (Findability, Accessibility, Interoperability and Reusability) data principles. A detailed logbook ensures to minimize the risks of data errors and inaccuracies as well as data sharing and reuse. Data set is password-protected, pseudonymized and coded for storage and analysis process. A safe and secure storage is provided and maintained by Linköping University. The collecting and processing of personal data from study participants is limited to data necessary to fulfil the objectives of the study. Transcriptions of audio interviews is checked by another person besides the transcriber. In all conversions, maintaining the original information content is ensured. Ethical committee statement and intellectual property rights regulates the storing and opening of research data.

PATIENT AND PUBLIC INVOLVEMENT

The project is run at *Barnafrid* – the Swedish National Center on Violence Against Children in collaboration with Save the Children Sweden and the Refugee Medical Centre. We have particularly chosen to include both public health and non-governmental sectors that can provide us with valuable up-to-date knowledge on the migrants in Sweden as well as to facilitate the dissemination of research findings and translation of the findings into coherent public health policies. No migrant children or young adults are directly involved in the study design, recruitment or conduct of the research. However, the results will be made available to participants, their caregivers, the funders, the professionals working with migrants and researchers.

ETHICS AND DISSEMINATION

Ethical aspects

Ethics approval has been granted by the Ethics Review Board of Linköping University (#2018-504-32) and the National Ethics Board (#2019-05473). Migrant children are considered a vulnerable group due to an increased risk of having been exposed to violence and it is sometimes suggested that asking about these experiences may cause distress. However, our

previous experiences have indicated that the risk for distress is small. Nevertheless, a plan has been established to take care of participants who need further support.

Participation is based on voluntary informed written consent. The participants, and their legal guardians if the participant is a minor, are informed about the purpose of the study, how the data is protected and stored and most importantly that the interview will have no bearing on their asylum process. They are also informed that they, at any time, can withdraw their consent and exit the study. Incentives valued at approximately €30 are given to adult participants.

To ensure that the project follows the stated ethical standards, weekly monitoring takes place and ethical issues are documented in a study log.

Knowledge dissemination and utilization plan

Information about the ongoing study and obtained results are communicated via a homepage (www.barnafrid.se/denlangaresan), lectures, podcasts, interviews and press releases as well as webinars in addition to social media (twitter @denlangaresan).

Community policymakers and stakeholders will be targeted separately using outreach events to implement new knowledge in current practices and to initiate improvements based on the results. For example, knowledge obtained in this project will be helpful to improve the existing screen of mental health problems and exposure to violence among migrants. Also, an intersectoral process development guide might be constructed to facilitate management and coordination of actions for prevention and intervention of mental health problems.

For peer review only

Authors` contributions

EM, AK and LK conceived the idea for the study protocol article. EM, AK, FF, LJ and LK designed the study. EM, AK and LK drafted the manuscript. All authors critically revised the work and approved the final submitted version.

Data availability

Data collection is ongoing. The results from this project will be published in open access journals. The data sets generated during the current study will be available from the corresponding author on reasonable request.

Funding

This study is funded by FORTE - the Swedish Research Council for Health, Working Life and Welfare (Grant ID 2019-01660) and was supported by a grant from the Queen Silvia`s foundation. None of the funders had no role in the designing the study, writing of the report and in the decision to submit the paper for publication.

Acknowledgements

Associate professor Gisela Priebe is acknowledged for contributing to the initial discussions on the study design.

Competing interests

The authors declare that they have no competing interests.

Patient consent for publication

Not required.

Ethics

This study has been approved Ethics Review Board of Linköping University (#2018-504-32) and the National Ethics Review Board (#2019-05473). Informed consent will be obtained from all study participants and legal guardians if the participant in younger than 15 years.

For peer review only

REFERENCES

1. United Nations High Commissioner for Refugees. Figures at a Glance 2019 [Available from: https://www.unhcr.org/figures-at-a-glance.html accessed 2020-08-14.

2. United Nations High Commissioner for Refugees. Global trends - Forced displacement in 2019 2020 [Available from: https://www.unhcr.org/statistics/unhcrstats/5ee200e37/unhcr-global-trends-2019.html accessed 2020-08-14.

3. Eurostat. Statistik om migration och migranter 2019 [Available from: https://ec.europa.eu/eurostat/statistics-explained/index.php?title=Migration_and_migrant_population_statistics/sv accessed 2020-08-14.

4. International Organization for Migration. World migration report 2017 [Available from: https://www.iom.int/wmr/2018 accessed 2020-08-14.

5. Migrationsverket. Asylsökande till Sverige 2000–2018 2019 [Available from: https://www.migrationsverket.se/Om-Migrationsverket/Statistik/Asyl.html accessed 2020-08-14.

6. European Council. European Council meeting (28 June 2018) –Conclusions. 2018 [Available from: https://www.consilium.europa.eu/en/meetings/european-council/2018/06/28-29/ accessed 2020-08-14.

7. Feijen L. The Challenges of Ensuring Protection to Unaccompanied and Separated Children in Composite Flows in Europe. *Refugee Survey Quarterly* 2008;27 doi: 10.1093/rsq/hdn049

8. Kadir A, Shenoda S, Goldhagen J. Effects of armed conflict on child health and development: A systematic review. *Plos One* 2019;14(1) doi: 10.1371/journal.pone.0210071

9. Lustig SL, Kia-Keating M, Knight WG, et al. Review of child and adolescent refugee mental health. *J Am Acad Child Adolesc Psychiatry* 2004;43(1):24-36. doi: 10.1097/00004583-200401000-00012 [published Online First: 2003/12/24]

10. Blackmore R, Gray KM, Boyle JA, et al. Systematic Review and Meta-Analysis: The Prevalence of Mental Illness in Child and Adolescent Refugees and Asylum Seekers. *Journal of the American Academy of Child & Adolescent Psychiatry* doi: 10.1016/j.jaac.2019.11.011

11. Jakobsen M, Demott MA, Heir T. Prevalence of psychiatric disorders among unaccompanied asylum-seeking adolescents in norway. *Clin Pract Epidemiol Ment*

For peer review only

Health 2014;10:53-8. doi: 10.2174/1745017901410010053 [published Online First: 2014/07/10]

12. Taleshan N, Petersen JH, Schioetz ML, et al. Multimorbidity and mortality thereof, among non-western refugees and family reunification immigrants in Denmark - a register based cohort study. *BMC Public Health* 2018;18(1):844. doi: 10.1186/s12889-018-5785-y [published Online First: 2018/07/08]
13. United Nations High Commissioner for Refugees. Europe - dead and missing at sea 2020 [Available from: https://data2.unhcr.org/en/dataviz/95 accessed 2020-08-14.
14. Barajas-Gonzalez RG, Ayón C, Torres F. Applying a Community Violence Framework to Understand the Impact of Immigration Enforcement Threat on Latino Children. *Social Policy Report* 2018;31(3):1-24. doi: 10.1002/sop2.1
15. Kien C, Sommer I, Faustmann A, et al. Prevalence of mental disorders in young refugees and asylum seekers in European Countries: a systematic review. *European Child and Adolescent Psychiatry* 2018 doi: 10.1007/s00787-018-1215-z
16. Vervliet M, Meyer Demott MA, Jakobsen M, et al. The mental health of unaccompanied refugee minors on arrival in the host country. *Scand J Psychol* 2014;55(1):33-7. doi: 10.1111/sjop.12094 [published Online First: 2014/08/19]
17. Fazel M, Reed RV, Panter-Brick C, et al. Mental health of displaced and refugee children resettled in high-income countries: Risk and protective factors. *The Lancet* 2012;379(9812):266-82. doi: 10.1016/S0140-6736(11)60051-2
18. Rutter M. Resilience as a dynamic concept. *Development and Psychopathology* 2012(24):335–44. doi: doi:10.1017/S0954579412000028
19. Fazel M, Wheeler J, Danesh J. Prevalence of serious mental disorder in 7000 refugees resettled in western countries: a systematic review. *Lancet* 2005;365(9467):1309-14. doi: 10.1016/s0140-6736(05)61027-6 [published Online First: 2005/04/13]
20. Felitti VJ, Anda RF, Nordenberg D, et al. Relationship of Childhood Abuse and Household Dysfunction to Many of the Leading Causes of Death in Adults: The Adverse Childhood Experiences (ACE) Study. *American journal of preventive medicine* 1998;14(4):245-58. doi: https://doi.org/10.1016/S0749-3797(98)00017-8
21. Jensen TK, Skar A-MS, Andersson ES, et al. Long-term mental health in unaccompanied refugee minors: pre- and post-flight predictors. *European Child & Adolescent Psychiatry* 2019;28(12):1671-82. doi: 10.1007/s00787-019-01340-6
22. Vervliet M, Lammertyn J, Broekaert E, et al. Longitudinal follow-up of the mental health of unaccompanied refugee minors.

For peer review only

23. Jakobsen M, DeMott MAM, Wentzel-Larsen T, et al. The impact of the asylum process on mental health: a longitudinal study of unaccompanied refugee minors in Norway. *Bmj Open* 2017;7(6) doi: 10.1136/bmjopen-2016-015157
24. Sheehan DV, Sheehan KH, Shytle RD, et al. Reliability and validity of the Mini International Neuropsychiatric Interview for Children and Adolescents (MINI-KID). *The Journal of clinical psychiatry* 2010;71(3):313-26. doi: 10.4088/JCP.09m05305whi [published Online First: 2010/03/25]
25. Sachser C, Berliner L, Holt T, et al. International development and psychometric properties of the Child and Adolescent Trauma Screen (CATS). *Journal of Affective Disorders* 2017;210 doi: 10.1016/j.jad.2016.12.040
26. Blevins CA, Weathers FW, Davis MT, et al. The Posttraumatic Stress Disorder Checklist for DSM-5 (PCL-5): Development and Initial Psychometric Evaluation. (1573-6598 (Electronic))
27. Finkelhor D, Hamby SL, Ormrod R, et al. The Juvenile Victimization Questionnaire: reliability, validity, and national norms. *Child Abuse Negl* 2005;29(4):383-412. doi: 10.1016/j.chiabu.2004.11.001 [published Online First: 2005/05/27]
28. Gartland D, Bond L, Olsson C, et al. Development of a multi-dimensional measure of resilience in adolescents: The Adolescent Resilience Questionnaire. *BMC medical research methodology* 2011;11:134. doi: 10.1186/1471-2288-11-134
29. Topp CW, Ostergaard SD, Sondergaard S, et al. The WHO-5 Well-Being Index: a systematic review of the literature. *Psychother Psychosom* 2015;84(3):167-76. doi: 10.1159/000376585 [published Online First: 2015/04/04]
30. Mundt JC, Marks IM, Shear MK, et al. The Work and Social Adjustment Scale: a simple measure of impairment in functioning. *British Journal of Psychiatry* 2002;180(5):461-64. doi: 10.1192/bjp.180.5.461 [published Online First: 2018/01/02]
31. American Psychiatric Association. Diagnostic and statistical manual of mental disorders (5th ed.)2013.
32. Hydén M. The teller-focused interview: Interviewing as a relational practice. *Qualitative Social Work* 2014;13(6):795-812. doi: 10.1177/1473325013506247
33. Braun V, Clarke V. Using thematic analysis in psychology. *Qualitative Research in Psychology* 2006;3(2):77-101. doi: 10.1191/1478088706qp063oa
34. Kristman-Valente A, Wells EA. The role of gender in the association between child maltreatment and substance use behavior: a systematic review of longitudinal research

For peer review only

from 1995 to 2011. *Substance use & misuse* 2013;48(8):645-60. doi: 10.3109/10826084.2013.800115 [published Online First: 2013/06/12]

For peer review only

FIGURES TEXTS

Figure 1. Summary of the study protocol.

Table 1. Data collection and used instruments.

Figure 2. Summary of the main exposures, outcomes, confounders and analysis as well as the expected impact of the study.

For peer review only

Table 1. Data collection and used instruments

Themes	Variable/measure	Method	Study group(s)
**Sociodemographics**	Age, sex, country of birth, self-identified ethnicity etc.	Semi-structured interview based on an interview guide	All except for those participating qualitative interviews
**SES**	Parents` education and occupation	Semi-structured interview based on an interview guide	See above
**Traumatic events**	Potentially traumatic events	JVQ	See above
**Mental health**	Psychiatric symptoms	MINI, MINI-KID	See above
	PTSD	CATS, PCL-5	See above
**Resilience**	Resilience in domains self, family, peers, school and society	ARQ	See above
**Impairment**	Observer rated functional capacity	GAF	See above
	Self-rating on impaired functioning	WSAS	See above
**Wellbeing**	Self-rating on physical health and well-being	WHO-5 wellbeing index	See above
**Social network**	Family members and contact with friends and family	Question regarding is the child lives with their family, the families where abouts and if and how they keep in contact.	See above
**Societal support**	Access to care and services	Semi-structured interview based on an interview guide	See above
**Biological data**	Genotype, biomarkers	Blood and saliva sampling and analysis	Young adult cohort and age and sex matched controls

JVQ, juvenile victimization questionnaire; MINI/MINI-KID, The MINI International Neuropsychiatric Interview; CATS, Child and Adolescent Trauma Screen; PCL-5, The PTSD Checklist for DSM-5; ARQ, Adolescent resilience questionnaire; GAF, Global assessment of functionality; WSAS, The work and adjustment scale.

For peer review only

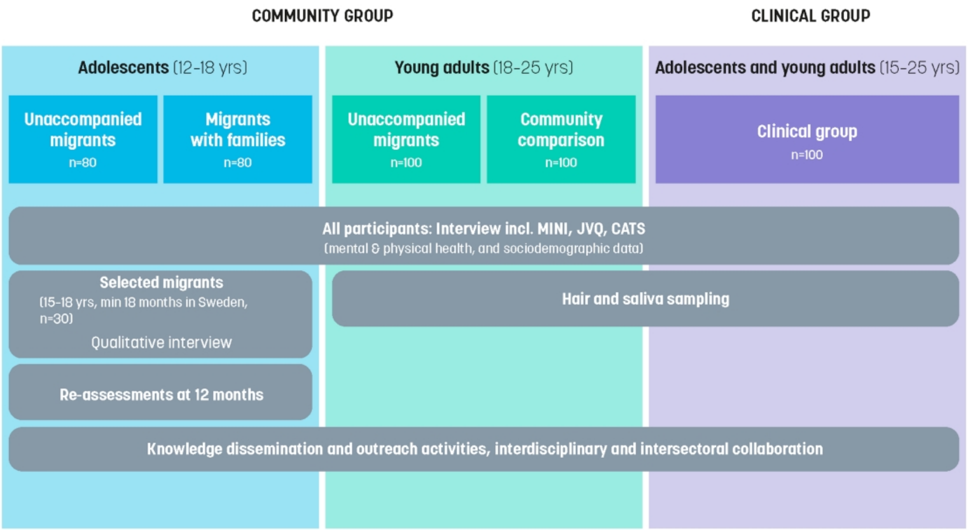

Figure 1. Summary of the study protocol.

261x147mm (300 x 300 DPI)

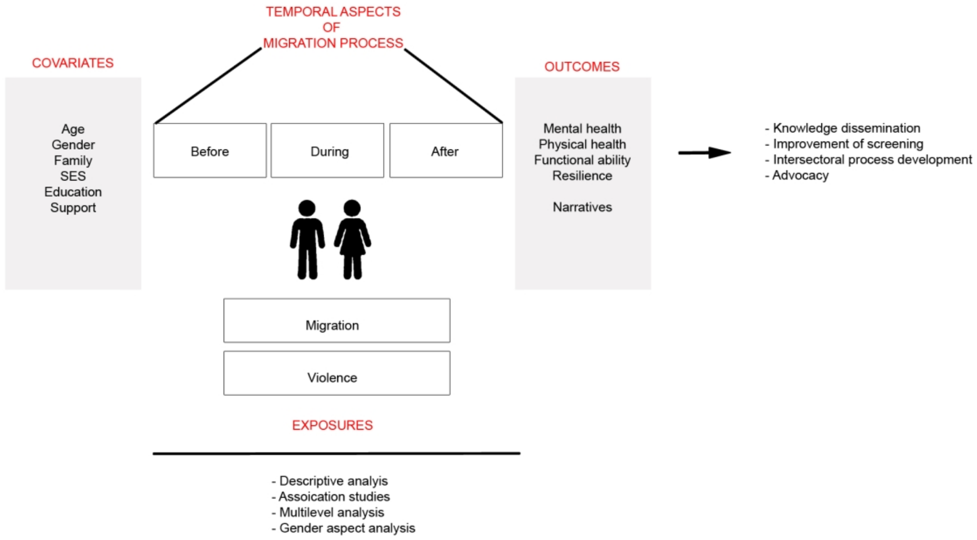

Figure 2. Summary of the main exposures, outcomes, confounders and analysis as well as the expected impact of the study.

BMJ Open

“The long journey to shelter” - study protocol: a prospective longitudinal analysis of mental health and its determinants, exposure to violence and subjective experiences of the migration process among adolescent and young adult migrants in Sweden

Journal:	*BMJ Open*
Manuscript ID	bmjopen-2020-043822.R1
Article Type:	Protocol
Date Submitted by the Author:	04-Jun-2021

Complete List of Authors:	Mattelin, Erica; Linköping University, Barnafrid, Department of Biomedical and Clinical Sciences<br>Khanolkar, Amal R ; University College London, Institute of Child Health; University College London, GOS Institute of Child Health<br>Fröberg, Frida; Linköping University, Barnafrid, Department of Biomedical and Clinical Sciences<br>Jonsson, Linda; Ersta Sköndal Bräcke University College<br>Korhonen, Laura; Linköping University, Barnafrid, Department of Child and Adolescent Psychiatry and Department of Biomedical and Clinical Sciences

**Primary Subject Heading**:	Mental health
Secondary Subject Heading:	Public health
Keywords:	MENTAL HEALTH, Child & adolescent psychiatry < PSYCHIATRY, PUBLIC HEALTH

SCHOLARONE™
Manuscripts

I, the Submitting Author has the right to grant and does grant on behalf of all authors of the Work (as defined in the below author licence), an exclusive licence and/or a non-exclusive licence for contributions from authors who are: i) UK Crown employees; ii) where BMJ has agreed a CC-BY licence shall apply, and/or iii) in accordance with the terms applicable for US Federal Government officers or employees acting as part of their official duties; on a worldwide, perpetual, irrevocable, royalty-free basis to BMJ Publishing Group Ltd ("BMJ") its licensees and where the relevant Journal is co-owned by BMJ to the co-owners of the Journal, to publish the Work in this journal and any other BMJ products and to exploit all rights, as set out in our licence.

The Submitting Author accepts and understands that any supply made under these terms is made by BMJ to the Submitting Author unless you are acting as an employee on behalf of your employer or a postgraduate student of an affiliated institution which is paying any applicable article publishing charge ("APC") for Open Access articles. Where the Submitting Author wishes to make the Work available on an Open Access basis (and intends to pay the relevant APC), the terms of reuse of such Open Access shall be governed by a Creative Commons licence – details of these licences and which Creative Commons licence will apply to this Work are set out in our licence referred to above.

Other than as permitted in any relevant BMJ Author's Self Archiving Policies, I confirm this Work has not been accepted for publication elsewhere, is not being considered for publication elsewhere and does not duplicate material already published. I confirm all authors consent to publication of this Work and authorise the granting of this licence.

For peer review only

"The long journey to shelter" - study protocol: a prospective longitudinal analysis of mental health and its determinants, exposure to violence and subjective experiences of the migration process among adolescent and young adult migrants in Sweden

Authors:

Erica Mattelin[1,2,3], Amal Khanolkar[4], Frida Fröberg[1], Linda S. Jonsson[5] and Laura Korhonen[1,2,6]

Affiliations:

[1] Barnafrid, Swedish National Center on Violence Against Children, Department of Biomedical and Clinical Sciences, Linköping University, Linköping, Sweden

[2] Center for Social and Affective Neuroscience, Department of Biomedical and Clinical Sciences, Linköping University, Linköping, Sweden

[3] Save the Children Sweden, Stockholm, Sweden

[4] MRC Unit for Lifelong Health & Ageing, University College of London, London, United Kingdom

[5] Ersta Sköndal Bräcke University College, Stockholm, Sweden

[6] Department of Child and Adolescent Psychiatry and Department of Biomedical and Clinical Sciences, Linköping University, Linköping, Sweden

Corresponding author: Professor Laura Korhonen, laura.korhonen@liu.se, telephone +46-13-28 11 81.

Running title: The long journey to shelter study protocol

Keywords: Migration, adolescent, young adult, mental health, violence, resilience, experience, biomarker, longitudinal study, health inequalities, ethnicity.

For peer review only

ABSTRACT

Introduction: According to the UN Refugee Agency (UNHCR), around 40% of the 79.5 million forcibly displaced persons in the end of the year 2019 were children. Exposure to violence and mental health problems such as posttraumatic stress disorder (PTSD) are frequently reported among migrant children but there is a knowledge gap in our understanding of the complex longitudinal interplay between individual, social and societal risk- and resilience factors that impact mental health and wellbeing, quality of life, and ability to function and adapt. There is also an urgent societal need to facilitate interdisciplinary and intersectoral collaborative efforts to develop effective methods to prevent, detect and respond to the needs of the migrants. This project will study adolescent and young adult migrants in Sweden using multiple methods such as quantitative analysis of data from a prospective cohort study and qualitative analysis of data gathered from teller-focused interviews. The aim is to understand how different factors impact mental health and integration into the Swedish society. Furthermore, individual experiences related to the migration process and exposure to violence will be studied in detail.

Methods and analysis: Study participants will include 490 migrants aged 12-25 years recruited through social services, health care, social media and the civil society. A subsample of adolescents (n=160) will be re-interviewed after one year. Data is collected using structured and semi-structured interviews along with saliva and hair sampling. Measures include sociodemographic data, longitudinal data on mental health and its determinants including genotypes and stress-hormone levels, access to health care and the process of migration including settlement in Sweden.

Ethics and dissemination: The Regional Ethics Board of Linköping (2018/292-31, 2018/504-32) and the National Ethics Board (2019-05473, 2020-00949) have approved the study. Results will be made available to participants, their caregivers, professionals working with migrants, researchers and the funders.

For peer review only

ARTICLE SUMMARY

Strengths and limitations of this study

- This is the first prospective longitudinal study in Sweden on newly arrived refugee and asylum seekeeing adolescents and young adults and aims to investigate exposure to violence, and mental and physical health using face-to-face interviews.
- Using a multimethod approach including analysis of genotypes and biomarkers, the study will provide a comprehensive set of data that allows a nuanced analysis of within immigrant population as well as context- and time-dependent factors that explain the different outcomes.
- Results can potentially improve care of migrants and aid in developing current intersectoral procedures including public health policies.
- Overrepresentation of study participants from particular countries could lead to selection bias.
- Loss to follow-up could impact the longitudinal analysis.

For peer review only

INTRODUCTION

Today millions of children are fleeing their countries, either voluntarily or being forced to. The group of migrant children is heterogeneous and includes asylum-seekers and refugees fleeing with their families as well as unaccompanied minors.[1] Most refugees/asylum seekers are forced to flee their homes due to acute threats such as conflicts, famine and instability but the migration problem also reflects massive socioeconomic inequalities that exist between different countries and regions.[2 3] Faced with this reality, migration is considered to be one of the greatest complex and current challenges that cannot be tackled quickly by simple measures.[4]

In 2019, of the 79.5 million people forced to flee their homes, 40% were minors.[2] During the peak immigration year of 2015, 70,384 children applied for asylum in Sweden; about half of them were unaccompanied.[5] Thereafter, the number of migrant children in Sweden has gradually decreased reflecting the European Union policy to limit migration.[5 6]

The definition of international migration encompasses the process of leaving the home country and settlement and integration into the society of destination. In practice, the process is most often intervened with complicated, stressful and potentially hazardous transient phases such as travelling in the hand of illegal smugglers, staying in multinational refugee camps or asylum accommodations and multiple replacements between different countries, accommodations and administrative systems with subsequent interruptions in established human relationships. Thus, migration is associated with several considerable risks such as being targeted by illegal smugglers and other types of crimes,[7] exposure to potentially traumatizing events as well as mental and physical health problems[8-11] including multimorbidity[12] and premature death.[13]

The arrival of migrants may be challenging for the host country. Barajas-Gonzalez et al [14] showed that immigration enforcement in the US leads to severe effects on children´s mental health, such as children living with a constant fear of their parents being taken into custody; leading the authors to suggest that the immigration enforcement may become a form of psychological violence. But in general, it is largely unexplored what damage is caused to children by slow and insecure bureaucracy.

Current state-of-the-art has shown that migrant children have experienced potentially traumatizing events more often than non-migrant children in high-income countries. Studies

also report a higher prevalence of mental health problems, such as PTSD, anxiety, and depression, among migrants compared to non-migrant children.[8 15 16] Several studies have demonstrated sex- or gender-based differences such as high rates of sexual violence against females[17-19]. However, the link between potentially traumatic events and mental health among migrant children has not been fully explained. This is largely because most studies till date have been cross-sectional in design, with small sample sizes and have focused on children that have already been in the host country for a considerable time.

Current research literature has largely focused on risk factors and mental illness.[20] Less attention has been paid to individual and social resilience factors and their interplay with risk factors which is important for, among other things, a favorable adaptation to a new environment[20 21]. Resilience entails the achievement of desirable social and emotional adaptation, although the individual was exposed to great risk[22]. Knowing that exposure to repeated violence is not only a direct danger to a child but also may have long-term consequences for the mental and physical health[23 24], efforts should be taken to strengthen the child's and family's resilience.

Objectives

The overall research aim is to study and understand how different factors impact health, wellbeing and integration of migrants in Sweden. We hypothesize that the refugee and asylum-seeking group of children is heterogeneous including well-adjusted individuals with good health and those with severe problems, multimorbidity and functional impairments. We further hypothesize that exposure to violence, lack of social support and network negatively impact mental health, functional ability and well-being.

This study aims:

- To identify individual-related factors that impact health and wellbeing. Includes studies on traumatization, mental health problems, resilience as well as genotypes, stress hormones and other biomarkers from collected biomaterial.
- To study which social factors that impact health, wellbeing and integration. Includes analysis of network and support.
- To describe children´s experiences related to the entire migration process.

In general, expected results from this study include estimates of the prevalence of experiences of different types of violence, polyvictimization, and mental health problems, as well as their impact on functional ability and wellbeing. Also, the roles played by social support as well as different biological factors in this context are elucidated. Qualitative studies are expected to give us information about how adolescents migrants conceptualize their own stories. Mechanisms of, among others, polyvictimization and resilience and their effects on health will be covered in future studies.

METHODS AND ANALYSIS

Design and participants

This study includes prospective longitudinal and cross-sectional designs. The term 'migrant' *("på flykt" in Swedish)* refers in this study to asylum seekers, refugees, family migrants, quota refugees and undocumented migrants. The population recruited to the study is migrant adolescents and young adults residing in Sweden during 2018-2021. As this is a heterogenous group, 4 different study groups will be included involving participants recruited from the community as well as from clinical settings (Figure 1).

1. *Adolescents (quantitative study):* 80 unaccompanied and 80 accompanied adolescent migrants that will be recruited in 2018 - 2021. Inclusion criteria: migrant, 12 – 18 years of age, and should not have applied for asylum in Sweden more than 6 months before the study interview. Follow-up interviews are done one year after the first interview.
2. *Adolescents (qualitative interviews)*: 30 adolescents for whom the inclusion criteria are 15-18 years of age, migrant, applied for asylum in Sweden at a maximum of 18 months before the interview date.
3. *Young adult migrants and Swedish born peers (quantitative study)*: This includes 100 young adults who migrated to Sweden after 2015. Inclusion criteria: migrants between the ages of 18 – 25 years living in Sweden. A comparison group consists of a sample of 100 young adults born in Sweden.
4. *Clinical sample (quantitative study)*: This will include 100 adolescent and young adults to be recruited from a trauma unit at a Refugee Medical Center in Norrköping, Sweden. Inclusion criteria: 15 – 25 years of age, migrant and living in Sweden.

National statistics on asylum seekers in Sweden is continuously followed to monitor the representativity of the all the subsamples.[25] If needed, targeted recruitments will be initiated.

For peer review only - http://bmjopen.bmj.com/site/about/guidelines.xhtml

Sample size

The aim is to collect data on 490 children and young adults. This sample size allows proposed analyses of primary outcomes with statistical strength of 0.80 and α <.05 and is based on previous studies with similar methodology [26 27 28] which included 95 - 138 adolescent migrants, with 47 - 77 adolescent migrants at follow-up assessments. We aim to have a larger sample to be able to do more detailed analyses.

Recruitment

Participants are recruited across Sweden since refugees/asylum-seekers are given a random municipality placement while waiting for a decision on their application for asylum. Recruitment is done via health care, social services, social media and the civil society such as non-governmental organizations working with the migrants. The clinical sample is recruited from the Refugee Medical Centre/Region Östergötland and the community comparison group mainly through high schools.

Recruitment of study participants is mainly via people working directly with refugee and asylum seekers who are given leaflets describing the study. Those who agree to participate will be subsequently contacted by the researchers to first obtain informed consent as well as to schedule an interview. All information is given in oral and written form in the participant`s native language.

Each interview is scheduled to last for two hours and a certified translator is used if needed. Interviews are held at a location chosen by the interviewee and/or the family.

Interviews

Semi-structured, translator assisted interviews are conducted to collect socio-demographic data on country of birth and self-identified ethnicity, family situation, schooling as well as social networks and social support (Table 1). Symptoms of mental disorders are assessed using the diagnostic interview MINI-KID/MINI.[29] Presence of PTSD is evaluated using the Child and Adolescent Trauma Screen [30] for those under 18 and PCL-5 for those over 18.[31] The Juvenile Victimization Questionnaire (JVQ) is used to measure the number of potentially traumatizing events.[32] The questionnaire has been adapted to this study to specifically assess the timing by including the question on whether potentially traumatizing events happened before, during or

For peer review only

after the process of fleeing one's country of origin. Also, additional questions on issues related to natural disasters, accidents, medical procedures, death of close relatives, poverty, separation from their parents, being captured or imprisoned and human trafficking are included. Resilience is evaluated using the Adolescent Resilience Questionnaire[33] including five different domains: self, family, peers, school and society. Participant will complete a self-rating on physical health and well-being using the WHO-5 wellbeing index.[34] The participants' functional capacity is mapped with the GAF and WSAS.[35 36] The interviews are verified by an experienced clinical psychologist to ensure good quality. Also, a random selection of 10% of the interviews are quality assessed independently by two members in the research group.

The qualitative interviews are conducted using the "Teller-focused interview" method which is particularly suitable for studies on sensitive topics and ongoing processes.[37] The interviews are semi-structured and include few overarching questions that covers areas such as the adolescents' experiences in the home country (e.g. school, friends, hobbies, wellbeing), issues related to the entire migration process (e.g. reasons to flee, memories from the journey, exposure to violence if any), life during the first 18 months in Sweden and their future aspirations for the 10 years ahead. Total time per assessment is approximately 60 minutes.

Biological material

A 3cm long hair sample is collected and stored at room temperature until analysis. Saliva samples are collected using Salivette Cortisol (Sarstedt AG & Co. KG, Nurnbrecht, Germany) and stored at -80° until analysis.

Data-analysis plan

Figure 2 provides an overview of the main exposures, outcomes, confounders and analysis that will be conducted. Descriptive analysis of collected data will include presenting continuous variables as mean values with standard deviations and categorical variables as proportions for the entire study sample but also by important covariates like sex, unaccompanied/accompanied and country of origin. Associations of interest between various variables will be analyzed using univariate linear regression or chi-square tests for differences of proportions for continuous and categorical variables, respectively.

A series of regression models will be performed to study associations between exposures (e.g. exposure to violence, asylum status) and outcomes of interest (e.g. mental health, functional

For peer review only

ability, comorbidity (for example co-occurrence of mental and physical health conditions) using multivariate linear and logistic regression for continuous and categorical outcomes respectively. Each set of regression models will be adjusted for potential confounders such as sex, family socioeconomic position, age and country of origin.

An analysis of individual, environmental and social factors that explain better adaptation, resilience, and level of function will be conducted. First, a descriptive analysis of the sociodemographic background, exposure to violence, the prevalence of mental and physical health problems, wellbeing and resilience factors, in the adolescent cohort and young adult sample will be performed. Second, the prevalence of violence, and mental health problems, and the association between violence and mental health problems in the different phases of the migration process in both groups will be estimated. Besides, the potential moderating effect of resilience factors on the association between exposure to violence and mental health problems in the adolescent cohort will be analyzed. Third, because it is assumed that the migration and resilience processes, and the development of mental health problems, are complex and potentially non-linear, multidirectional and multilevel, we will also conduct a multilevel analysis of longitudinal data.

Qualitative interviews will be analyzed according to the principles for thematic analysis.[38] Genotypes and biomarkers such as stress hormone levels and immunoprofiles will be analyzed using standard methods.

Gender aspects

We know, based on previous research that the associations between certain risk factors, such as traumatic events, and mental health differs between the sexes.[39] Such differences are often attributed to genetic or biological factors, and sociological processes, such as conforming to gender normative behavior or how girls and boys are educated differently, for example. When it comes to the experiences and health of migrant children, less effort has been made to understand the importance of gender. In our study, we will initially stratify analyses by sex and potential sex differences will then be interpreted with a gender perspective; with the intention of understanding and explaining the importance of gender in the link between exposure and outcome. We will also take into account that our respondents may choose to identify themselves by ethnicity, sexuality, family socioeconomic background.

For peer review only - http://bmjopen.bmj.com/site/about/guidelines.xhtml

Methodological issues

The longitudinal study design will allow analysis of changes over time in the same study participant, providing stronger evidence for causality than could be obtained from a cross-sectional design. Combined interview and biological data will provide a versatile set of information that can be used to address different kinds of research questions. Use of standardized structured and semi-structured interviews and validated instruments will also allow comparisons to other studies.

The primary limitation of this study may arise if migrants from a few countries with a high certainty of getting asylum such as Syria will be overrepresented in the study. Furthermore, we could also have the risk of concerned parents or social workers not letting all the adolescents participate because of fear of causing distress. This may exclude certain groups such as those with existing mental health problems and/or exposed to violence.

Yet another potential limitation is the loss to follow-up because migrants might leave the country. Efforts to minimize loss to follow-up will include respecting the time commitment of patients, formal consented tracking procedures of patients enrolled, multiple contacts for arranging follow-up and flexible hours and places for interviews.

Data management, storage and security

Throughout the course of this project, research data is collected and processed according to FAIR (Findability, Accessibility, Interoperability and Reusability) data principles. A detailed logbook ensures to minimize the risks of data errors and inaccuracies as well as data sharing and reuse. Data set is password-protected, pseudonymized and coded for storage and analysis process. A safe and secure storage is provided and maintained by Linköping University. The collecting and processing of personal data from study participants is limited to data necessary to fulfil the objectives of the study. Transcriptions of audio interviews is checked by another person besides the transcriber. In all conversions, maintaining the original information content is ensured. Ethical committee statement and intellectual property rights regulates the storing and opening of research data.

PATIENT AND PUBLIC INVOLVEMENT

The project is based at *Barnafrid* – the Swedish National Center on Violence Against Children in collaboration with Save the Children Sweden and the Refugee Medical Centre. We have

For peer review

particularly chosen to include both public health and non-governmental sectors that can provide us with valuable up-to-date knowledge on the migrants in Sweden as well as to facilitate the dissemination of research findings and translation of the findings into coherent public health policies. No migrant children or young adults are directly involved in the study design, recruitment or conduct of the research. However, the results will be made available to participants, their caregivers, the funders, the professionals working with migrants and researchers.

ETHICS AND DISSEMINATION

Ethical aspects

Ethics approval has been granted by the Regional Ethics Board of Linköping (2018/292-31, 2018/504-32) and the National Ethics Board (2019-05473, 2020-00949). Migrant children are considered a vulnerable group due to an increased risk of having been exposed to violence and it is sometimes suggested that asking about these experiences may cause distress. However, our previous experiences have indicated that the risk for distress is small. Nevertheless, a plan has been established to take care of participants who need further support.

Participation is based on voluntary informed written consent. The participants, and their legal guardians if the participant is a minor, are informed about the purpose of the study, how the data is protected and stored and most importantly that the interview will have no bearing on their asylum process. They are also informed that they, at any time, can withdraw their consent and exit the study. Incentives valued at approximately €30 are given to adult participants.

To ensure that the project follows the stated ethical standards, weekly monitoring takes place and ethical issues are documented in a study log.

Knowledge dissemination and utilization plan

Information about the ongoing study and obtained results are communicated via a homepage (www.barnafrid.se/denlangaresan), lectures, podcasts, interviews and press releases as well as webinars in addition to social media (twitter@denlangaresan).

Community policymakers and stakeholders will be targeted separately using outreach events to implement new knowledge in current practices and to initiate improvements based on the results. For example, knowledge obtained in this project will be helpful to improve the existing

For peer review only

screen of mental health problems and exposure to violence among migrants. Also, an intersectoral process development guide might be constructed to facilitate management and coordination of actions for prevention and intervention of mental health problems.

For peer review only

Authors` contributions

EM, AK and LK conceived the idea for the study protocol article. EM, AK, FF, LJ and LK designed the study. EM, AK and LK drafted the manuscript. All authors critically revised the work and approved the final submitted version.

Data availability

Data collection is ongoing. The results from this project will be published in open access journals. The data sets generated during the current study will be available from the corresponding author on reasonable request.

Funding

This study is funded by FORTE - the Swedish Research Council for Health, Working Life and Welfare (Grant ID 2019-01660) and was supported by a grant from the Queen Silvia`s foundation. None of the funders had no role in the designing the study, writing of the report and in the decision to submit the paper for publication.

Acknowledgements

Professor Gisela Priebe is acknowledged for contributing to the initial discussions on the study design.

Competing interests

The authors declare that they have no competing interests.

Patient consent for publication

Not required.

Ethics

This study has been approved the Regional Ethics Board of Linköping (2018/292-31, 2018/504-32) and the National Ethics Board (2019-05473, 2020-00949). Informed consent will be obtained from all study participants and legal guardians if the participant in younger than 15 years.

For peer review only

REFERENCES

1. United Nations High Commissioner for Refugees. Figures at a Glance 2019 [Available from: https://www.unhcr.org/figures-at-a-glance.html accessed 2020-08-14.
2. United Nations High Commissioner for Refugees. Global trends - Forced displacement in 2019 2020 [Available from: https://www.unhcr.org/statistics/unhcrstats/5ee200e37/unhcr-global-trends-2019.html accessed 2020-08-14.
3. Eurostat. Statistik om migration och migranter 2019 [Available from: https://ec.europa.eu/eurostat/statistics-explained/index.php?title=Migration_and_migrant_population_statistics/sv accessed 2020-08-14.
4. International Organization for Migration. World migration report 2017 [Available from: https://www.iom.int/wmr/2018 accessed 2020-08-14.
5. Migrationsverket. Asylsökande till Sverige 2000–2018 2019 [Available from: https://www.migrationsverket.se/Om-Migrationsverket/Statistik/Asyl.html accessed 2020-08-14.
6. European Council. European Council meeting (28 June 2018) –Conclusions. 2018 [Available from: https://www.consilium.europa.eu/en/meetings/european-council/2018/06/28-29/ accessed 2020-08-14.
7. Feijen L. The Challenges of Ensuring Protection to Unaccompanied and Separated Children in Composite Flows in Europe. *Refugee Survey Quarterly* 2008;27 doi: 10.1093/rsq/hdn049
8. Kadir A, Shenoda S, Goldhagen J. Effects of armed conflict on child health and development: A systematic review. *Plos One* 2019;14(1) doi: 10.1371/journal.pone.0210071
9. Lustig SL, Kia-Keating M, Knight WG, et al. Review of child and adolescent refugee mental health. *J Am Acad Child Adolesc Psychiatry* 2004;43(1):24-36. doi: 10.1097/00004583-200401000-00012 [published Online First: 2003/12/24]
10. Blackmore R, Gray KM, Boyle JA, et al. Systematic Review and Meta-Analysis: The Prevalence of Mental Illness in Child and Adolescent Refugees and Asylum Seekers. *Journal of the American Academy of Child & Adolescent Psychiatry* doi: 10.1016/j.jaac.2019.11.011
11. Jakobsen M, Demott MA, Heir T. Prevalence of psychiatric disorders among unaccompanied asylum-seeking adolescents in norway. *Clin Pract Epidemiol Ment Health* 2014;10:53-8. doi: 10.2174/1745017901410010053 [published Online First: 2014/07/10]
12. Taleshan N, Petersen JH, Schioetz ML, et al. Multimorbidity and mortality thereof, among non-western refugees and family reunification immigrants in Denmark - a register based cohort study. *BMC Public Health* 2018;18(1):844. doi: 10.1186/s12889-018-5785-y [published Online First: 2018/07/08]
13. United Nations High Commissioner for Refugees. Europe - dead and missing at sea 2020 [Available from: https://data2.unhcr.org/en/dataviz/95 accessed 2020-08-14.
14. Barajas-Gonzalez RG, Ayón C, Torres F. Applying a Community Violence Framework to Understand the Impact of Immigration Enforcement Threat on Latino Children. *Social Policy Report* 2018;31(3):1-24. doi: 10.1002/sop2.1
15. Kien C, Sommer I, Faustmann A, et al. Prevalence of mental disorders in young refugees and asylum seekers in European Countries: a systematic review. *European Child and Adolescent Psychiatry* 2018 doi: 10.1007/s00787-018-1215-z
16. Vervliet M, Meyer Demott MA, Jakobsen M, et al. The mental health of unaccompanied refugee minors on arrival in the host country. *Scand J Psychol* 2014;55(1):33-7. doi: 10.1111/sjop.12094 [published Online First: 2014/08/19]
17. Ward J, Vann B. Gender-based violence in refugee settings. *The Lancet* 2002;360:s13-s14. doi: 10.1016/S0140-6736(02)11802-2
18. Vu A, Adam A, Wirtz A, et al. The Prevalence of Sexual Violence among Female Refugees in Complex Humanitarian Emergencies: a Systematic Review and Meta-analysis. *PLoS Curr*

For peer review only

2014;6:ecurrents.dis.835f10778fd80ae031aac12d3b533ca7. doi: 10.1371/currents.dis.835f10778fd80ae031aac12d3b533ca7
19. Araujo JdO, Souza FMd, Proença R, et al. Prevalence of sexual violence among refugees: a systematic review. *Revista de saude publica* 2019;53:78.
20. Fazel M, Reed RV, Panter-Brick C, et al. Mental health of displaced and refugee children resettled in high-income countries: Risk and protective factors. *The Lancet* 2012;379(9812):266-82. doi: 10.1016/S0140-6736(11)60051-2
21. Gottvall M, Vaez M, Saboonchi F. Social support attenuates the link between torture exposure and post-traumatic stress disorder among male and female Syrian refugees in Sweden. *BMC International Health and Human Rights* 2019;19(1):28. doi: 10.1186/s12914-019-0214-6
22. Rutter M. Resilience as a dynamic concept. *Development and Psychopathology* 2012(24):335–44. doi: doi:10.1017/S0954579412000028
23. Gilbert R, Widom CS, Browne K, et al. Burden and consequences of child maltreatment in high-income countries. *The Lancet* 2009;373(9657):68-81. doi: 10.1016/S0140-6736(08)61706-7
24. Felitti VJ, Anda RF, Nordenberg D, et al. Relationship of Childhood Abuse and Household Dysfunction to Many of the Leading Causes of Death in Adults: The Adverse Childhood Experiences (ACE) Study. *American journal of preventive medicine* 1998;14(4):245-58. doi: https://doi.org/10.1016/S0749-3797(98)00017-8
25. Migrationsverket. Statistics 2021 [Available from: https://www.migrationsverket.se/English/About-the-Migration-Agency/Statistics.html.
26. Jensen TK, Skar A-MS, Andersson ES, et al. Long-term mental health in unaccompanied refugee minors: pre- and post-flight predictors. *European Child & Adolescent Psychiatry* 2019;28(12):1671-82. doi: 10.1007/s00787-019-01340-6
27. Vervliet M, Lammertyn J, Broekaert E, et al. Longitudinal follow-up of the mental health of unaccompanied refugee minors.
28. Jakobsen M, DeMott MAM, Wentzel-Larsen T, et al. The impact of the asylum process on mental health: a longitudinal study of unaccompanied refugee minors in Norway. *Bmj Open* 2017;7(6) doi: 10.1136/bmjopen-2016-015157
29. Sheehan DV, Sheehan KH, Shytle RD, et al. Reliability and validity of the Mini International Neuropsychiatric Interview for Children and Adolescents (MINI-KID). *The Journal of clinical psychiatry* 2010;71(3):313-26. doi: 10.4088/JCP.09m05305whi [published Online First: 2010/03/25]
30. Sachser C, Berliner L, Holt T, et al. International development and psychometric properties of the Child and Adolescent Trauma Screen (CATS). *Journal of Affective Disorders* 2017;210 doi: 10.1016/j.jad.2016.12.040
31. Blevins CA, Weathers FW, Davis MT, et al. The Posttraumatic Stress Disorder Checklist for DSM-5 (PCL-5): Development and Initial Psychometric Evaluation. (1573-6598 (Electronic))
32. Finkelhor D, Hamby SL, Ormrod R, et al. The Juvenile Victimization Questionnaire: reliability, validity, and national norms. *Child Abuse Negl* 2005;29(4):383-412. doi: 10.1016/j.chiabu.2004.11.001 [published Online First: 2005/05/27]
33. Gartland D, Bond L, Olsson C, et al. Development of a multi-dimensional measure of resilience in adolescents: The Adolescent Resilience Questionnaire. *BMC medical research methodology* 2011;11:134. doi: 10.1186/1471-2288-11-134
34. Topp CW, Ostergaard SD, Sondergaard S, et al. The WHO-5 Well-Being Index: a systematic review of the literature. *Psychother Psychosom* 2015;84(3):167-76. doi: 10.1159/000376585 [published Online First: 2015/04/04]
35. Mundt JC, Marks IM, Shear MK, et al. The Work and Social Adjustment Scale: a simple measure of impairment in functioning. *British Journal of Psychiatry* 2002;180(5):461-64. doi: 10.1192/bjp.180.5.461 [published Online First: 2018/01/02]
36. American Psychiatric Association. Diagnostic and statistical manual of mental disorders (5th ed.)2013.

For peer review only

37. Hydén M. The teller-focused interview: Interviewing as a relational practice. *Qualitative Social Work* 2014;13(6):795-812. doi: 10.1177/1473325013506247
38. Braun V, Clarke V. Using thematic analysis in psychology. *Qualitative Research in Psychology* 2006;3(2):77-101. doi: 10.1191/1478088706qp063oa
39. Kristman-Valente A, Wells EA. The role of gender in the association between child maltreatment and substance use behavior: a systematic review of longitudinal research from 1995 to 2011. *Substance use & misuse* 2013;48(8):645-60. doi: 10.3109/10826084.2013.800115 [published Online First: 2013/06/12]

For peer review only

FIGURES TEXTS

Figure 1. Summary of the study protocol.

Table 1. Data collection and used instruments.

Figure 2. Summary of the main exposures, outcomes, confounders and analysis as well as the expected impact of the study.

For peer review only

Table 1. Data collection and used instruments

Themes	Variable/measure	Method	Study group(s)
**Sociodemographics**	Age, sex, country of birth, self-identified ethnicity etc.	Semi-structured interview based on an interview guide	All except for those participating qualitative interviews
**SES**	Parents` education and occupation	Semi-structured interview based on an interview guide	See above
**Traumatic events**	Potentially traumatic events	JVQ	See above
**Mental health**	Psychiatric symptoms	MINI, MINI-KID	See above
	PTSD	CATS, PCL-5	See above
**Resilience**	Resilience in domains self, family, peers, school and society	ARQ	See above
**Impairment**	Observer rated functional capacity	GAF	See above
	Self-rating on impaired functioning	WSAS	See above
**Wellbeing**	Self-rating on physical health and well-being	WHO-5 wellbeing index	See above
**Social network**	Family members and contact with friends and family	Question regarding is the child lives with their family, the families where abouts and if and how they keep in contact.	See above
**Societal support**	Access to care and services	Semi-structured interview based on an interview guide	See above
**Biological data**	Genotype, biomarkers	Blood and saliva sampling and analysis	Young adult cohort and age and sex matched controls

JVQ, juvenile victimization questionnaire; MINI/MINI-KID, The MINI International Neuropsychiatric Interview; CATS, Child and Adolescent Trauma Screen; PCL-5, The PTSD Checklist for DSM-5; ARQ, Adolescent resilience questionnaire; GAF, Global assessment of functionality; WSAS, The work and adjustment scale.

For peer review only

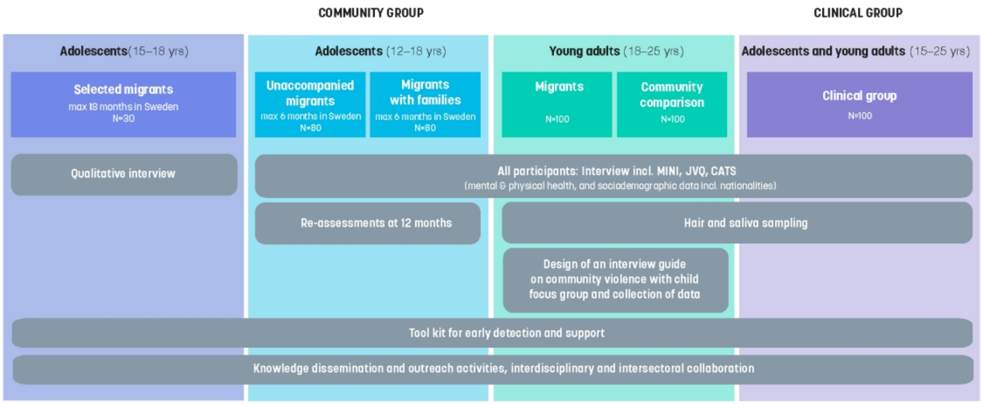

Figure 1. Summary of the study protocol.

261x147mm (200 x 200 DPI)

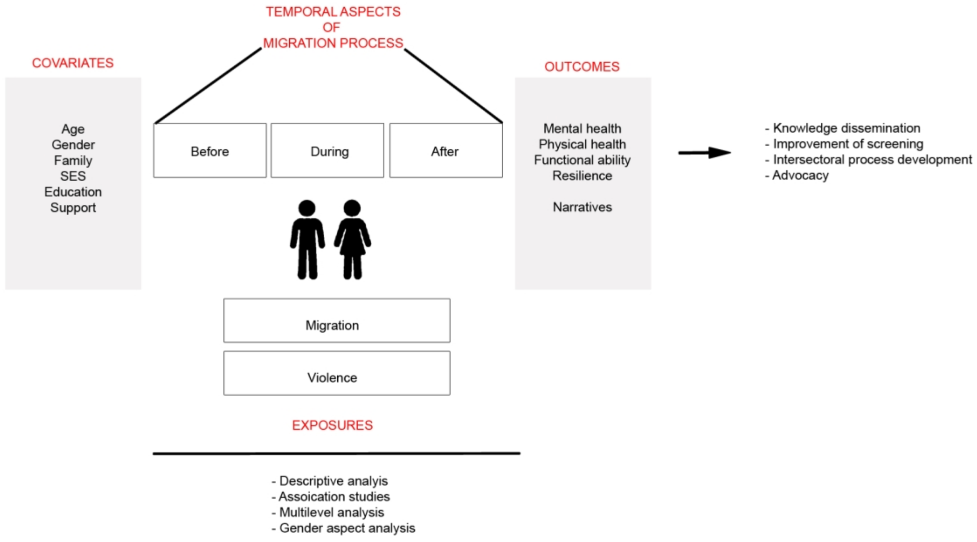

Figure 2. Summary of the main exposures, outcomes, confounders and analysis as well as the expected impact of the study.

BMJ Open

“The long journey to shelter” - study protocol: a prospective longitudinal analysis of mental health and its determinants, exposure to violence and subjective experiences of the migration process among adolescent and young adult migrants in Sweden

Journal:	*BMJ Open*
Manuscript ID	bmjopen-2020-043822.R2
Article Type:	Protocol
Date Submitted by the Author:	12-Sep-2021

Complete List of Authors:	Mattelin, Erica; Linköping University, Barnafrid, Department of Biomedical and Clinical Sciences<br>Khanolkar, Amal R ; University College London, Institute of Child Health; University College London, GOS Institute of Child Health<br>Fröberg, Frida; Linköping University, Barnafrid, Department of Biomedical and Clinical Sciences<br>Jonsson, Linda; Ersta Sköndal Bräcke University College<br>Korhonen, Laura; Linköping University, Barnafrid, Department of Child and Adolescent Psychiatry and Department of Biomedical and Clinical Sciences

**Primary Subject Heading**:	Mental health
Secondary Subject Heading:	Public health
Keywords:	MENTAL HEALTH, Child & adolescent psychiatry < PSYCHIATRY, PUBLIC HEALTH

SCHOLARONE™ Manuscripts

I, the Submitting Author has the right to grant and does grant on behalf of all authors of the Work (as defined in the below author licence), an exclusive licence and/or a non-exclusive licence for contributions from authors who are: i) UK Crown employees; ii) where BMJ has agreed a CC-BY licence shall apply, and/or iii) in accordance with the terms applicable for US Federal Government officers or employees acting as part of their official duties; on a worldwide, perpetual, irrevocable, royalty-free basis to BMJ Publishing Group Ltd ("BMJ") its licensees and where the relevant Journal is co-owned by BMJ to the co-owners of the Journal, to publish the Work in this journal and any other BMJ products and to exploit all rights, as set out in our licence.

The Submitting Author accepts and understands that any supply made under these terms is made by BMJ to the Submitting Author unless you are acting as an employee on behalf of your employer or a postgraduate student of an affiliated institution which is paying any applicable article publishing charge ("APC") for Open Access articles. Where the Submitting Author wishes to make the Work available on an Open Access basis (and intends to pay the relevant APC), the terms of reuse of such Open Access shall be governed by a Creative Commons licence – details of these licences and which Creative Commons licence will apply to this Work are set out in our licence referred to above.

Other than as permitted in any relevant BMJ Author's Self Archiving Policies, I confirm this Work has not been accepted for publication elsewhere, is not being considered for publication elsewhere and does not duplicate material already published. I confirm all authors consent to publication of this Work and authorise the granting of this licence.

For peer review only

"The long journey to shelter" - study protocol: a prospective longitudinal analysis of mental health and its determinants, exposure to violence and subjective experiences of the migration process among adolescent and young adult migrants in Sweden

Authors:

Erica Mattelin[1,2,3], Amal Khanolkar[4], Frida Fröberg[1], Linda S. Jonsson[5] and Laura Korhonen[1,2,6]

Affiliations:

[1] Barnafrid, Swedish National Center on Violence Against Children, Department of Biomedical and Clinical Sciences, Linköping University, Linköping, Sweden

[2] Center for Social and Affective Neuroscience, Department of Biomedical and Clinical Sciences, Linköping University, Linköping, Sweden

[3] Save the Children Sweden, Stockholm, Sweden

[4] MRC Unit for Lifelong Health & Ageing, University College of London, London, United Kingdom

[5] Ersta Sköndal Bräcke University College, Stockholm, Sweden

[6] Department of Child and Adolescent Psychiatry and Department of Biomedical and Clinical Sciences, Linköping University, Linköping, Sweden

Corresponding author: Professor Laura Korhonen, laura.korhonen@liu.se, telephone +46-13-28 11 81.

Running title: The long journey to shelter study protocol

Keywords: Migration, adolescent, young adult, mental health, violence, resilience, experience, biomarker, longitudinal study, health inequalities, ethnicity.

For peer review only

ABSTRACT

Introduction: According to the UN Refugee Agency (UNHCR), around 40% of the 79.5 million forcibly displaced persons in the end of the year 2019 were children. Exposure to violence and mental health problems such as posttraumatic stress disorder (PTSD) are frequently reported among migrant children but there is a knowledge gap in our understanding of the complex longitudinal interplay between individual, social and societal risk- and resilience factors that impact mental health and wellbeing, quality of life, and ability to function and adapt. There is also an urgent societal need to facilitate interdisciplinary and intersectoral collaborative efforts to develop effective methods to prevent, detect and respond to the needs of the migrants. This project will study adolescent and young adult migrants in Sweden using multiple methods such as quantitative analysis of data from a prospective cohort study and qualitative analysis of data gathered from teller-focused interviews. The aim is to understand how different factors impact mental health and integration into the Swedish society. Furthermore, individual experiences related to the migration process and exposure to violence will be studied in detail.

Methods and analysis: Study participants will include 490 migrants aged 12-25 years recruited through social services, health care, social media and the civil society. A subsample of adolescents (n=160) will be re-interviewed after one year. Data is collected using structured and semi-structured interviews along with saliva and hair sampling. Measures include sociodemographic data, longitudinal data on mental health and its determinants including genotypes and stress-hormone levels, access to health care and the process of migration including settlement in Sweden.

Ethics and dissemination: The Regional Ethics Board of Linköping (2018/292-31, 2018/504-32) and the National Ethics Board (2019-05473, 2020-00949) have approved the study. Results will be made available to participants, their caregivers, professionals working with migrants, researchers and the funders.

For peer review only

ARTICLE SUMMARY

Strengths and limitations of this study

- This is the first prospective longitudinal study in Sweden on newly arrived refugee and asylum seekeeing adolescents and young adults and aims to investigate exposure to violence, and mental and physical health using face-to-face interviews. However, this protocol will not address cultural, religious, economic, or political factors due to limits of possible data collection in interpreter-assisted interviews.
- Using a multimethod approach including analysis of genotypes and biomarkers, the study will provide a comprehensive set of data that allows a nuanced analysis of within immigrant population as well as context- and time-dependent factors that explain the different outcomes.
- Results can potentially improve care of migrants and aid in developing current intersectoral procedures including public health policies.
- Overrepresentation of study participants from particular countries could lead to selection bias.
- Loss to follow-up could impact the longitudinal analysis.

For peer review only

INTRODUCTION

Today millions of children are fleeing their countries, either voluntarily or being forced to. The group of migrant children is heterogeneous and includes asylum-seekers and refugees fleeing with their families as well as unaccompanied minors.[1] Most refugees/asylum seekers are forced to flee their homes due to acute threats such as conflicts, famine and instability but the migration problem also reflects massive socioeconomic inequalities that exist between different countries and regions.[2 3] Faced with this reality, migration is considered to be one of the greatest complex and current challenges that cannot be tackled quickly by simple measures.[4]

In 2019, of the 79.5 million people forced to flee their homes, 40% were minors.[2] During the peak immigration year of 2015, 70,384 children applied for asylum in Sweden; about half of them were unaccompanied.[5] Thereafter, the number of migrant children in Sweden has gradually decreased reflecting the European Union policy to limit migration.[5 6] In 2020, approximately 13 000 asylum seekers, including 3566 children, came to Sweden. Most of the asylum seeking children originated from Syria, Afghanistan, Iraq, and Eritrea.[7]

The definition of international migration encompasses the process of leaving the home country and settlement and integration into the society of destination. In practice, the process is most often intervened with complicated, stressful and potentially hazardous transient phases such as travelling in the hand of illegal smugglers, staying in multinational refugee camps or asylum accommodations and multiple replacements between different countries, accommodations and administrative systems with subsequent interruptions in established human relationships. Thus, migration is associated with several considerable risks such as being targeted by illegal smugglers and other types of crimes,[8] exposure to potentially traumatizing events as well as mental and physical health problems[9-12] including multimorbidity[13] and premature death.[14]

The arrival of migrants may be challenging for the host country. Barajas-Gonzalez et al [15] showed that immigration enforcement in the US leads to severe effects on children´s mental health, such as children living with a constant fear of their parents being taken into custody; leading the authors to suggest that the immigration enforcement may become a form of psychological violence. But in general, it is largely unexplored what damage is caused to children by slow and insecure bureaucracy.

For peer review only - http://bmjopen.bmj.com/site/about/guidelines.xhtml

Current state-of-the-art has shown that migrant children have experienced potentially traumatizing events more often than non-migrant children in high-income countries. Studies also report a higher prevalence of mental health problems, such as PTSD, anxiety, and depression, among migrants compared to non-migrant children.[9 16 17] Several studies have demonstrated sex- or gender-based differences such as high rates of sexual violence against females[18-20]. However, the link between potentially traumatic events and mental health among migrant children has not been fully explained. This is largely because most studies till date have been cross-sectional in design, with small sample sizes and have focused on children that have already been in the host country for a considerable time.

Current research literature has largely focused on risk factors and mental illness.[21] Less attention has been paid to individual and social resilience factors and their interplay with risk factors which is important for, among other things, a favorable adaptation to a new environment[21 22]. Resilience entails the achievement of desirable social and emotional adaptation, although the individual was exposed to great risk[23]. Knowing that exposure to repeated violence is not only a direct danger to a child but also may have long-term consequences for the mental and physical health[24 25], efforts should be taken to strengthen the child's and family's resilience.

Objectives

The overall research aim is to study and understand how different factors impact health, wellbeing and integration of migrants in Sweden. We hypothesize that the refugee and asylum-seeking group of children is heterogeneous including well-adjusted individuals with good health and those with severe problems, multimorbidity and functional impairments. We further hypothesize that exposure to violence, lack of social support and network negatively impact mental health, functional ability and well-being.

This study aims:

- To identify individual-related factors that impact health and wellbeing. Includes studies on traumatization, mental health problems, resilience as well as genotypes, stress hormones and other biomarkers from collected biomaterial.
- To study which social factors that impact health, wellbeing and integration. Includes analysis of network and support.
- To describe children´s experiences related to the entire migration process.

In general, expected results from this study include estimates of the prevalence of experiences of different types of violence, polyvictimization, and mental health problems, as well as their impact on functional ability and wellbeing. Also, the roles played by social support as well as different biological factors in this context are elucidated. Qualitative studies are expected to give us information about how adolescents migrants conceptualize their own stories. Mechanisms of, among others, polyvictimization and resilience and their effects on health will be covered in future studies.

METHODS AND ANALYSIS

Design and participants

The term 'migrant' *("på flykt" in Swedish)* refers to refugees (i.e. those defined according to the 1951 Refugee convention), asylum seekers, family reunification migrants (i.e. family to a person with a permanent- or temporary residence permit), quota refugees (i.e. a person who has been selected by the UNHCR to be resettled to a third country) and undocumented migrants (i.e. former asylum-seekers who lack proper authorization to stay). The rationale for including all categories of refugees is to obtain a holistic picture of the current situation as well as to be able to follow similarities and differences between groups. The population recruited to the study is migrant adolescents and young adults residing in Sweden during 2018-2021. As this is a heterogenous group, 4 different study groups will be included involving participants recruited from the community as well as from clinical settings (Figure 1).

1 *Adolescents (quantitative study):* 80 unaccompanied and 80 accompanied adolescent migrants that will be recruited in 2018 - 2021. Inclusion criteria: migrant, 12 – 18 years of age, and should not have applied for asylum in Sweden more than 6 months before the study interview. Follow-up interviews are done one year after the first interview.

2 *Adolescents (qualitative interviews)*: 30 adolescents for whom the inclusion criteria are 15-18 years of age, migrant, applied for asylum in Sweden at a maximum of 18 months before the interview date.

3 *Young adult migrants and Swedish born peers (quantitative study)*: This includes 100 young adults who migrated to Sweden after 2015. Inclusion criteria: migrants between the ages of 18 – 25 years living in Sweden. A comparison group consists of a sample of 100 young adults born in Sweden.

For peer review only

4 *Clinical sample (quantitative study)*: This will include 100 adolescent and young adults to be recruited from a trauma unit at a Refugee Medical Center in Norrköping, Sweden. Inclusion criteria: 15 – 25 years of age, migrant and living in Sweden.

National statistics on asylum seekers in Sweden is continuously followed to monitor the representativity of the all the subsamples.[26] If needed, targeted recruitments will be initiated.

Sample size

The aim is to collect data on 490 children and young adults. This sample size allows proposed analyses of primary outcomes with statistical strength of 0.80 and $\alpha < .05$ and is based on previous studies with similar methodology [27] [28] [29] which included 95 - 138 adolescent migrants, with 47 - 77 adolescent migrants at follow-up assessments. We aim to have a larger sample to be able to do more detailed analyses.

Recruitment

Participants are recruited across Sweden since refugees/asylum-seekers are given a random municipality placement while waiting for a decision on their application for asylum. Recruitment is done via health care, social services, social media and the civil society such as non-governmental organizations working with the migrants. The clinical sample is recruited from the Refugee Medical Centre/Region Östergötland and the community comparison group mainly through high schools.

Recruitment of study participants is mainly via people working directly with refugee and asylum seekers who are given leaflets describing the study. Those who agree to participate will be subsequently contacted by the researchers to first obtain informed consent as well as to schedule an interview. All information is given in oral and written form in the participant`s native language.

Each interview is scheduled to last for two hours and a certified translator is used if needed. Interviews are held at a location chosen by the interviewee and/or the family.

Interviews

Semi-structured, translator assisted interviews are conducted to collect socio-demographic data on country of birth and self-identified ethnicity, family situation, schooling as well as

For peer review only

social networks and social support (Table 1). Symptoms of mental disorders are assessed using the diagnostic interview MINI-KID/MINI.[30] Presence of PTSD is evaluated using the Child and Adolescent Trauma Screen [31] for those under 18 and PCL-5 for those over 18.[32] The Juvenile Victimization Questionnaire (JVQ) is used to measure the number of potentially traumatizing events.[33] The questionnaire has been adapted to this study to specifically assess the timing by including the question on whether potentially traumatizing events happened before, during or after the process of fleeing one's country of origin. Also, additional questions on issues related to natural disasters, accidents, medical procedures, death of close relatives, poverty, separation from their parents, being captured or imprisoned and human trafficking are included. Resilience is evaluated using the Adolescent Resilience Questionnaire[34] including five different domains: self, family, peers, school and society. Participant will complete a self-rating on physical health and well-being using the WHO-5 wellbeing index.[35] The participants' functional capacity is mapped with the GAF and WSAS.[36 37] The interviews are verified by an experienced clinical psychologist to ensure good quality. Also, a random selection of 10% of the interviews are quality assessed independently by two members in the research group.

The qualitative interviews are conducted using the "Teller-focused interview" method which is particularly suitable for studies on sensitive topics and ongoing processes.[38] The interviews are semi-structured and include few overarching questions that covers areas such as the adolescents' experiences in the home country (e.g. school, friends, hobbies, wellbeing), issues related to the entire migration process (e.g. reasons to flee, memories from the journey, exposure to violence if any), life during the first 18 months in Sweden and their future aspirations for the 10 years ahead. Total time per assessment is approximately 60 minutes.

Biological material

A 3cm long hair sample is collected and stored at room temperature until analysis. Saliva samples are collected using Salivette Cortisol (Sarstedt AG & Co. KG, Nurnbrecht, Germany) and stored at -80° until analysis.

Data-analysis plan

Figure 2 provides an overview of the main exposures, outcomes, confounders and analysis that will be conducted. Descriptive analysis of collected data will include presenting continuous variables as mean values with standard deviations and categorical variables as proportions for the entire study sample but also by important covariates like sex, unaccompanied/accompanied

For peer review only

and country of origin. Associations of interest between various variables will be analyzed using univariate linear regression or chi-square tests for differences of proportions for continuous and categorical variables, respectively.

A series of regression models will be performed to study associations between exposures (e.g. exposure to violence, asylum status) and outcomes of interest (e.g. mental health, functional ability, comorbidity (for example co-occurrence of mental and physical health conditions) using multivariate linear and logistic regression for continuous and categorical outcomes respectively. Each set of regression models will be adjusted for potential confounders such as sex, family socioeconomic position, age and country of origin.

An analysis of individual, environmental and social factors that explain better adaptation, resilience, and level of function will be conducted. First, a descriptive analysis of the sociodemographic background, exposure to violence, the prevalence of mental and physical health problems, wellbeing and resilience factors, in the adolescent cohort and young adult sample will be performed. Second, the prevalence of violence, and mental health problems, and the association between violence and mental health problems in the different phases of the migration process in both groups will be estimated. Besides, the potential moderating effect of resilience factors on the association between exposure to violence and mental health problems in the adolescent cohort will be analyzed. Third, because it is assumed that the migration and resilience processes, and the development of mental health problems, are complex and potentially non-linear, multidirectional and multilevel, we will also conduct a multilevel analysis of longitudinal data.

Qualitative interviews will be analyzed according to the principles for thematic analysis.[39] Genotypes and biomarkers such as stress hormone levels and immunoprofiles will be analyzed using standard methods.

Gender aspects

We know, based on previous research that the associations between certain risk factors, such as traumatic events, and mental health differs between the sexes.[40] Such differences are often attributed to genetic or biological factors, and sociological processes, such as conforming to gender normative behavior or how girls and boys are educated differently, for example. When it comes to the experiences and health of migrant children, less effort has been made to

For peer review only

understand the importance of gender. In our study, we will initially stratify analyses by sex and potential sex differences will then be interpreted with a gender perspective; with the intention of understanding and explaining the importance of gender in the link between exposure and outcome. We will also take into account that our respondents may choose to identify themselves by ethnicity, sexuality, family socioeconomic background.

Methodological issues

The longitudinal study design will allow analysis of changes over time in the same study participant, providing stronger evidence for causality than could be obtained from a cross-sectional design. Combined interview and biological data will provide a versatile set of information that can be used to address different kinds of research questions. Use of standardized structured and semi-structured interviews and validated instruments will also allow comparisons to other studies.

The primary limitation of this study may arise if migrants from a few countries with a high certainty of getting asylum such as Syria will be overrepresented in the study. Furthermore, we could also have the risk of concerned parents or social workers not letting all the adolescents participate because of fear of causing distress. This may exclude certain groups such as those with existing mental health problems and/or exposed to violence.

Yet another potential limitation is the loss to follow-up because migrants might leave the country. Efforts to minimize loss to follow-up will include respecting the time commitment of patients, formal consented tracking procedures of patients enrolled, multiple contacts for arranging follow-up and flexible hours and places for interviews.

Data management, storage and security

Throughout the course of this project, research data is collected and processed according to FAIR (Findability, Accessibility, Interoperability and Reusability) data principles. A detailed logbook ensures to minimize the risks of data errors and inaccuracies as well as data sharing and reuse. Data set is password-protected, pseudonymized and coded for storage and analysis process. A safe and secure storage is provided and maintained by Linköping University. The collecting and processing of personal data from study participants is limited to data necessary to fulfil the objectives of the study. Transcriptions of audio interviews is checked by another person besides the transcriber. In all conversions, maintaining the original information content

For peer review only

is ensured. Ethical committee statement and intellectual property rights regulates the storing and opening of research data.

PATIENT AND PUBLIC INVOLVEMENT

The project is based at *Barnafrid* – the Swedish National Center on Violence Against Children in collaboration with Save the Children Sweden and the Refugee Medical Centre. We have particularly chosen to include both public health and non-governmental sectors that can provide us with valuable up-to-date knowledge on the migrants in Sweden as well as to facilitate the dissemination of research findings and translation of the findings into coherent public health policies. No migrant children or young adults are directly involved in the study design, recruitment or conduct of the research. However, the results will be made available to participants, their caregivers, the funders, the professionals working with migrants and researchers.

ETHICS AND DISSEMINATION

Ethical aspects

Ethics approval has been granted by the Regional Ethics Board of Linköping (2018/292-31, 2018/504-32) and the National Ethics Board (2019-05473, 2020-00949). Migrant children are considered a vulnerable group due to an increased risk of having been exposed to violence and it is sometimes suggested that asking about these experiences may cause distress. However, our previous experiences have indicated that the risk for distress is small. Nevertheless, a plan has been established to take care of participants who need further support.

Participation is based on voluntary informed written consent. The participants, and their legal guardians if the participant is a minor, are informed about the purpose of the study, how the data is protected and stored and most importantly that the interview will have no bearing on their asylum process. They are also informed that they, at any time, can withdraw their consent and exit the study. Incentives valued at approximately €30 are given to adult participants.

To ensure that the project follows the stated ethical standards, weekly monitoring takes place and ethical issues are documented in a study log.

Knowledge dissemination and utilization plan

For peer review only

Information about the ongoing study and obtained results are communicated via a homepage (www.barnafrid.se/denlangaresan), lectures, podcasts, interviews and press releases as well as webinars in addition to social media (twitter@denlangaresan).

Community policymakers and stakeholders will be targeted separately using outreach events to implement new knowledge in current practices and to initiate improvements based on the results. For example, knowledge obtained in this project will be helpful to improve the existing screen of mental health problems and exposure to violence among migrants. Also, an intersectoral process development guide might be constructed to facilitate management and coordination of actions for prevention and intervention of mental health problems.

For peer review only

Authors` contributions

EM, AK and LK conceived the idea for the study protocol article. EM, AK, FF, LJ and LK designed the study. EM, AK and LK drafted the manuscript. All authors critically revised the work and approved the final submitted version.

Data availability

Data collection is ongoing. The results from this project will be published in open access journals. The data sets generated during the current study will be available from the corresponding author on reasonable request.

Funding

This study is funded by FORTE - the Swedish Research Council for Health, Working Life and Welfare (Grant ID 2019-01660) and was supported by a grant from the Queen Silvia`s foundation. None of the funders had no role in the designing the study, writing of the report and in the decision to submit the paper for publication.

Acknowledgements

Professor Gisela Priebe is acknowledged for contributing to the initial discussions on the study design.

Competing interests

The authors declare that they have no competing interests.

Patient consent for publication

Not required.

Ethics

This study has been approved the Regional Ethics Board of Linköping (2018/292-31, 2018/504-32) and the National Ethics Board (2019-05473, 2020-00949). Informed consent will be obtained from all study participants and legal guardians if the participant in younger than 15 years.

For peer review only

REFERENCES

1. United Nations High Commissioner for Refugees. Figures at a Glance 2019 [Available from: https://www.unhcr.org/figures-at-a-glance.html accessed 2020-08-14

.

2. United Nations High Commissioner for Refugees. Global trends - Forced displacement in 2019 2020 [Available from: https://www.unhcr.org/statistics/unhcrstats/5ee200e37/unhcr-global-trends-2019.html accessed 2020-08-14.
3. Eurostat. Statistik om migration och migranter 2019 [Available from: https://ec.europa.eu/eurostat/statistics-explained/index.php?title=Migration_and_migrant_population_statistics/sv accessed 2020-08-14.
4. International Organization for Migration. World migration report 2017 [Available from: https://www.iom.int/wmr/2018 accessed 2020-08-14.
5. Migrationsverket. Asylsökande till Sverige 2000–2018 2019 [Available from: https://www.migrationsverket.se/Om-Migrationsverket/Statistik/Asyl.html accessed 2020-08-14.
6. European Council. European Council meeting (28 June 2018) –Conclusions. 2018 [Available from: https://www.consilium.europa.eu/en/meetings/european-council/2018/06/28-29/ accessed 2020-08-14.
7. Migrationsverket. Asyl: Över-sikter och statistik från tidi-gare peri-oder 2021 [accessed 2021-02-14.
8. Feijen L. The Challenges of Ensuring Protection to Unaccompanied and Separated Children in Composite Flows in Europe. *Refugee Survey Quarterly* 2008;27 doi: 10.1093/rsq/hdn049
9. Kadir A, Shenoda S, Goldhagen J. Effects of armed conflict on child health and development: A systematic review. *Plos One* 2019;14(1) doi: 10.1371/journal.pone.0210071
10. Lustig SL, Kia-Keating M, Knight WG, et al. Review of child and adolescent refugee mental health. *J Am Acad Child Adolesc Psychiatry* 2004;43(1):24-36. doi: 10.1097/00004583-200401000-00012 [published Online First: 2003/12/24]
11. Blackmore R, Gray KM, Boyle JA, et al. Systematic Review and Meta-Analysis: The Prevalence of Mental Illness in Child and Adolescent Refugees and Asylum Seekers. *Journal of the American Academy of Child & Adolescent Psychiatry* doi: 10.1016/j.jaac.2019.11.011
12. Jakobsen M, Demott MA, Heir T. Prevalence of psychiatric disorders among unaccompanied asylum-seeking adolescents in norway. *Clin Pract Epidemiol Ment Health* 2014;10:53-8. doi: 10.2174/1745017901410010053 [published Online First: 2014/07/10]
13. Taleshan N, Petersen JH, Schioetz ML, et al. Multimorbidity and mortality thereof, among non-western refugees and family reunification immigrants in Denmark - a register based cohort study. *BMC Public Health* 2018;18(1):844. doi: 10.1186/s12889-018-5785-y [published Online First: 2018/07/08]
14. United Nations High Commissioner for Refugees. Europe - dead and missing at sea 2020 [Available from: https://data2.unhcr.org/en/dataviz/95 accessed 2020-08-14.
15. Barajas-Gonzalez RG, Ayón C, Torres F. Applying a Community Violence Framework to Understand the Impact of Immigration Enforcement Threat on Latino Children. *Social Policy Report* 2018;31(3):1-24. doi: 10.1002/sop2.1
16. Kien C, Sommer I, Faustmann A, et al. Prevalence of mental disorders in young refugees and asylum seekers in European Countries: a systematic review. *European Child and Adolescent Psychiatry* 2018 doi: 10.1007/s00787-018-1215-z
17. Vervliet M, Meyer Demott MA, Jakobsen M, et al. The mental health of unaccompanied refugee minors on arrival in the host country. *Scand J Psychol* 2014;55(1):33-7. doi: 10.1111/sjop.12094 [published Online First: 2014/08/19]
18. Ward J, Vann B. Gender-based violence in refugee settings. *The Lancet* 2002;360:s13-s14. doi: 10.1016/S0140-6736(02)11802-2

For peer review only

19. Vu A, Adam A, Wirtz A, et al. The Prevalence of Sexual Violence among Female Refugees in Complex Humanitarian Emergencies: a Systematic Review and Meta-analysis. *PLoS Curr* 2014;6:ecurrents.dis.835f10778fd80ae031aac12d3b533ca7. doi: 10.1371/currents.dis.835f10778fd80ae031aac12d3b533ca7
20. Araujo JdO, Souza FMd, Proença R, et al. Prevalence of sexual violence among refugees: a systematic review. *Revista de saude publica* 2019;53:78.
21. Fazel M, Reed RV, Panter-Brick C, et al. Mental health of displaced and refugee children resettled in high-income countries: Risk and protective factors. *The Lancet* 2012;379(9812):266-82. doi: 10.1016/S0140-6736(11)60051-2
22. Gottvall M, Vaez M, Saboonchi F. Social support attenuates the link between torture exposure and post-traumatic stress disorder among male and female Syrian refugees in Sweden. *BMC International Health and Human Rights* 2019;19(1):28. doi: 10.1186/s12914-019-0214-6
23. Rutter M. Resilience as a dynamic concept. *Development and Psychopathology* 2012(24):335–44. doi: doi:10.1017/S0954579412000028
24. Gilbert R, Widom CS, Browne K, et al. Burden and consequences of child maltreatment in high-income countries. *The Lancet* 2009;373(9657):68-81. doi: 10.1016/S0140-6736(08)61706-7
25. Felitti VJ, Anda RF, Nordenberg D, et al. Relationship of Childhood Abuse and Household Dysfunction to Many of the Leading Causes of Death in Adults: The Adverse Childhood Experiences (ACE) Study. *American journal of preventive medicine* 1998;14(4):245-58. doi: https://doi.org/10.1016/S0749-3797(98)00017-8
26. Migrationsverket. Statistics 2021 [Available from: https://www.migrationsverket.se/English/About-the-Migration-Agency/Statistics.html.
27. Jensen TK, Skar A-MS, Andersson ES, et al. Long-term mental health in unaccompanied refugee minors: pre- and post-flight predictors. *European Child & Adolescent Psychiatry* 2019;28(12):1671-82. doi: 10.1007/s00787-019-01340-6
28. Vervliet M, Lammertyn J, Broekaert E, et al. Longitudinal follow-up of the mental health of unaccompanied refugee minors.
29. Jakobsen M, DeMott MAM, Wentzel-Larsen T, et al. The impact of the asylum process on mental health: a longitudinal study of unaccompanied refugee minors in Norway. *Bmj Open* 2017;7(6) doi: 10.1136/bmjopen-2016-015157
30. Sheehan DV, Sheehan KH, Shytle RD, et al. Reliability and validity of the Mini International Neuropsychiatric Interview for Children and Adolescents (MINI-KID). *The Journal of clinical psychiatry* 2010;71(3):313-26. doi: 10.4088/JCP.09m05305whi [published Online First: 2010/03/25]
31. Sachser C, Berliner L, Holt T, et al. International development and psychometric properties of the Child and Adolescent Trauma Screen (CATS). *Journal of Affective Disorders* 2017;210 doi: 10.1016/j.jad.2016.12.040
32. Blevins CA, Weathers FW, Davis MT, et al. The Posttraumatic Stress Disorder Checklist for DSM-5 (PCL-5): Development and Initial Psychometric Evaluation. (1573-6598 (Electronic))
33. Finkelhor D, Hamby SL, Ormrod R, et al. The Juvenile Victimization Questionnaire: reliability, validity, and national norms. *Child Abuse Negl* 2005;29(4):383-412. doi: 10.1016/j.chiabu.2004.11.001 [published Online First: 2005/05/27]
34. Gartland D, Bond L, Olsson C, et al. Development of a multi-dimensional measure of resilience in adolescents: The Adolescent Resilience Questionnaire. *BMC medical research methodology* 2011;11:134. doi: 10.1186/1471-2288-11-134
35. Topp CW, Ostergaard SD, Sondergaard S, et al. The WHO-5 Well-Being Index: a systematic review of the literature. *Psychother Psychosom* 2015;84(3):167-76. doi: 10.1159/000376585 [published Online First: 2015/04/04]
36. Mundt JC, Marks IM, Shear MK, et al. The Work and Social Adjustment Scale: a simple measure of impairment in functioning. *British Journal of Psychiatry* 2002;180(5):461-64. doi: 10.1192/bjp.180.5.461 [published Online First: 2018/01/02]

For peer review only

37. American Psychiatric Association. Diagnostic and statistical manual of mental disorders (5th ed.)2013.
38. Hydén M. The teller-focused interview: Interviewing as a relational practice. *Qualitative Social Work* 2014;13(6):795-812. doi: 10.1177/1473325013506247
39. Braun V, Clarke V. Using thematic analysis in psychology. *Qualitative Research in Psychology* 2006;3(2):77-101. doi: 10.1191/1478088706qp063oa
40. Kristman-Valente A, Wells EA. The role of gender in the association between child maltreatment and substance use behavior: a systematic review of longitudinal research from 1995 to 2011. *Substance use & misuse* 2013;48(8):645-60. doi: 10.3109/10826084.2013.800115 [published Online First: 2013/06/12]

For peer review only

FIGURES TEXTS

Figure 1. Summary of the study protocol.

Table 1. Data collection and used instruments.

Figure 2. Summary of the main exposures, outcomes, confounders and analysis as well as the expected impact of the study.

For peer review only

Table 1. Data collection and used instruments

Themes	Variable/measure	Method	Study group(s)
**Sociodemographics**	Age, sex, country of birth, self-identified ethnicity etc.	Semi-structured interview based on an interview guide	All except for those participating qualitative interviews
**SES**	Parents` education and occupation	Semi-structured interview based on an interview guide	See above
**Traumatic events**	Potentially traumatic events	JVQ	See above
**Mental health**	Psychiatric symptoms	MINI, MINI-KID	See above
	PTSD	CATS, PCL-5	See above
**Resilience**	Resilience in domains self, family, peers, school and society	ARQ	See above
**Impairment**	Observer rated functional capacity	GAF	See above
	Self-rating on impaired functioning	WSAS	See above
**Wellbeing**	Self-rating on physical health and well-being	WHO-5 wellbeing index	See above
**Social network**	Family members and contact with friends and family	Question regarding is the child lives with their family, the families where abouts and if and how they keep in contact.	See above
**Societal support**	Access to care and services	Semi-structured interview based on an interview guide	See above
**Biological data**	Genotype, biomarkers	Blood and saliva sampling and analysis	Young adult cohort and age and sex matched controls

JVQ, juvenile victimization questionnaire; MINI/MINI-KID, The MINI International Neuropsychiatric Interview; CATS, Child and Adolescent Trauma Screen; PCL-5, The PTSD Checklist for DSM-5; ARQ, Adolescent resilience questionnaire; GAF, Global assessment of functionality; WSAS, The work and adjustment scale.

For peer review only

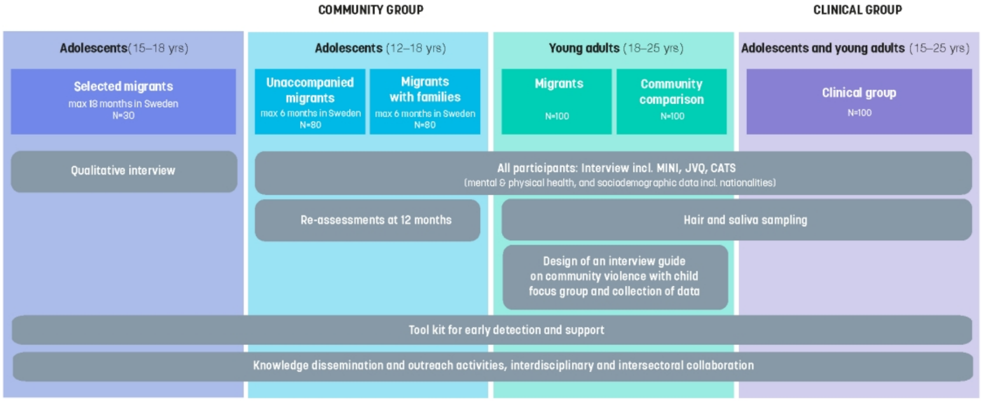

Figure 1. Summary of the study protocol.

261x147mm (200 x 200 DPI)

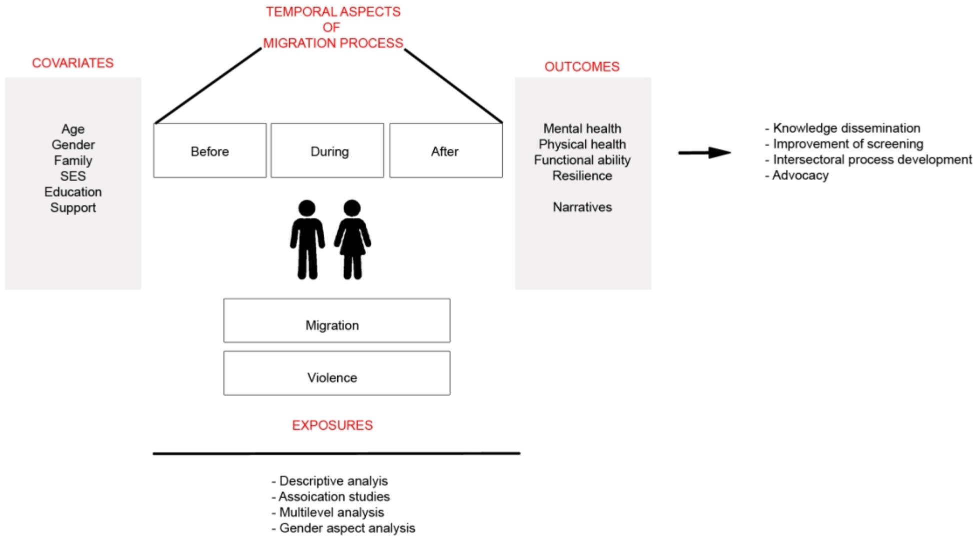

Figure 2. Summary of the main exposures, outcomes, confounders and analysis as well as the expected impact of the study.
